# Supplementary material for: Single cell transcriptomics reveals opioid usage evokes widespread suppression of antiviral gene program
Source: Nat Commun. 2020 May 26;11:2611. doi: 10.1038/s41467-020-16159-y (PMC7250875; doi:10.1038/s41467-020-16159-y)
Supplement: Supplementary file 1 — Supplementary Information [file 41467_2020_16159_MOESM1_ESM.pdf]

Single cell transcriptomics reveals opioid usage evokes widespread suppression of antiviral gene program

Karagiannis and Cleary et al.

Supplementary Information

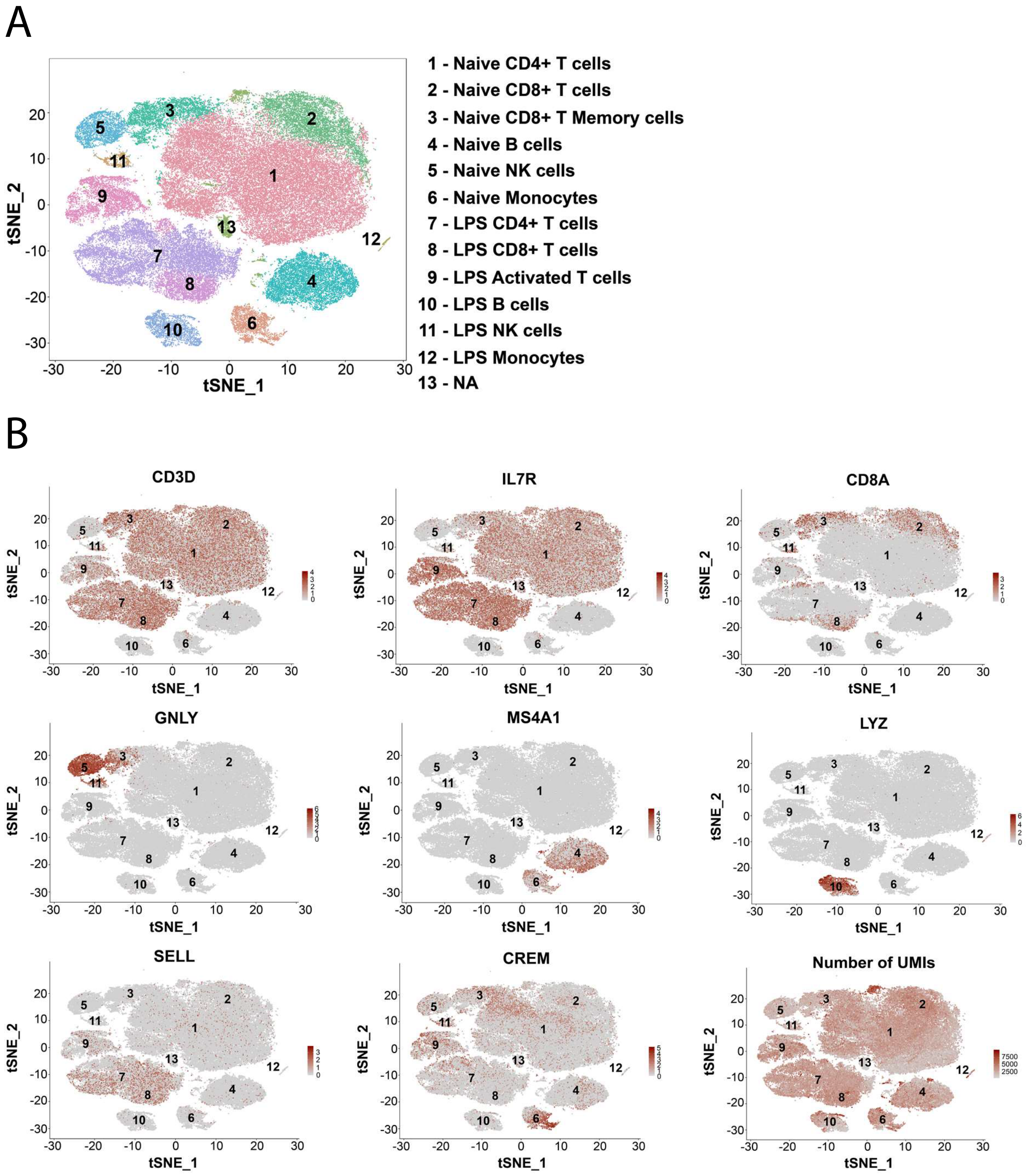

Supplementary Figure 1. Cell type identification from scRNA-seq of naive and LPS treated PBMCs from opioid-dependent individuals and non-dependent controls using canonical gene markers. a, t-SNE plot of naive and LPS treated PBMCs with identified cell types: Naive CD4+ T cells (30,384 cells), Naive CD8+ T cells (6,429 cells), Naive CD8+ memory T cells (3,579 cells), Naive B cells (6,692 cells), Naive NK cells (2,427 cells), Naive monocytes (1971 cells), LPS treated: CD4+ T cells (11,307 cells), LPS treated: CD8+ T cells (3,019 cells), LPS treated: activated T cells (4,056 cells), LPS treated: B cells (2,225 cells), LPS treated: NK cells (566 cells), LPS treated: monocytes (259 cells), NA or no applicable cell types (1,418 cells). **b**, t-SNE projection of canonical gene marker expression and number of UMIs per cell across all naive state and LPS treated subpopulations: CD4+ T cells (CD3D, IL7R), CD8+ T cells (CD8A), B cells (MS4A1), NK cells (GNLY), Monocytes (LYZ), activated cells (CREM), naive cells (SELL).

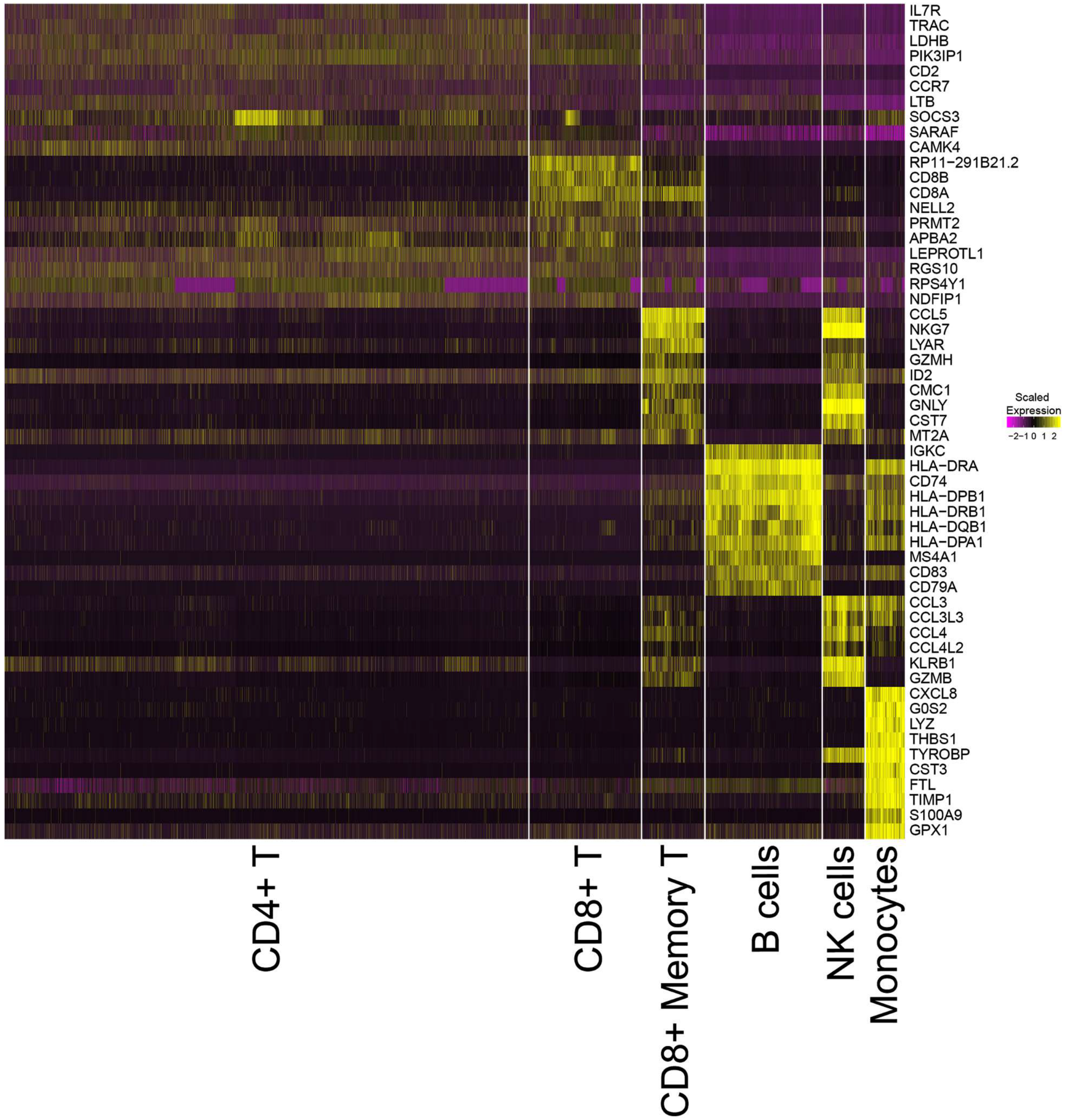

Supplementary Figure 2. Top marker genes for naive state PBMC cell types. Heatmap of the top 10 genes expressed (scaled) across cells in each naive state population. Top 10 marker genes were identified using a two-sided wilcoxon rank sum test over all cell types: CD4+ T cells (CD4+ T), CD8+ T cells (CD8+ T), CD8+ memory T cells (CD8+ T Memory), B cells, NK cells, and Monocytes.

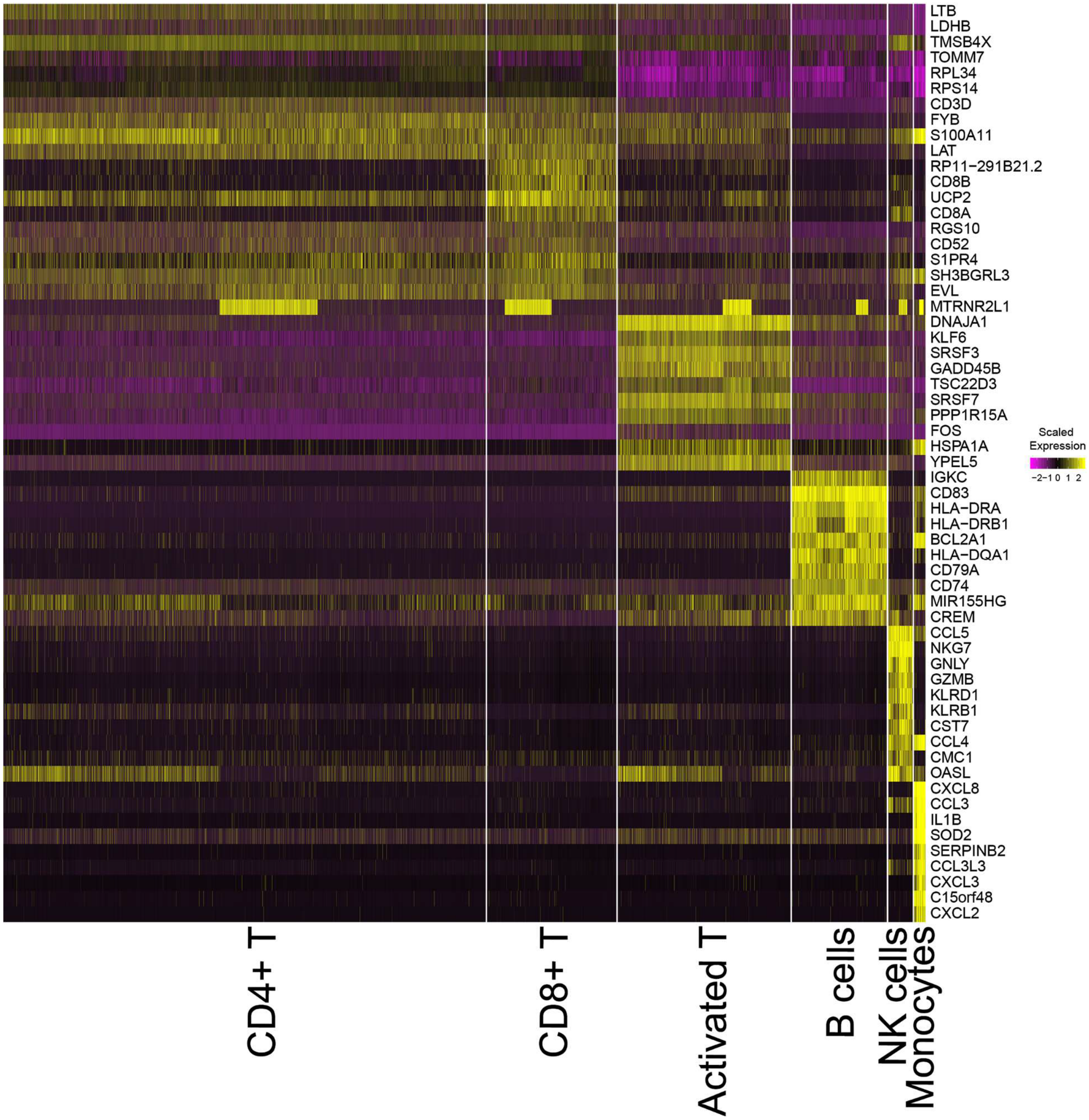

Supplementary Figure 3. Top marker genes for LPS treated PBMC cell types. Heatmap of the top 10 gene markers expressed (scaled) across cells in each LPS treated population. Top 10 marker genes were identified using a two-sided wilcoxon rank sum test over all cell types: CD4+ T cells (CD4+ T), CD8+ T cells (CD8+ T), activated T cells (Activated T), B cells, NK cells, and Monocytes.

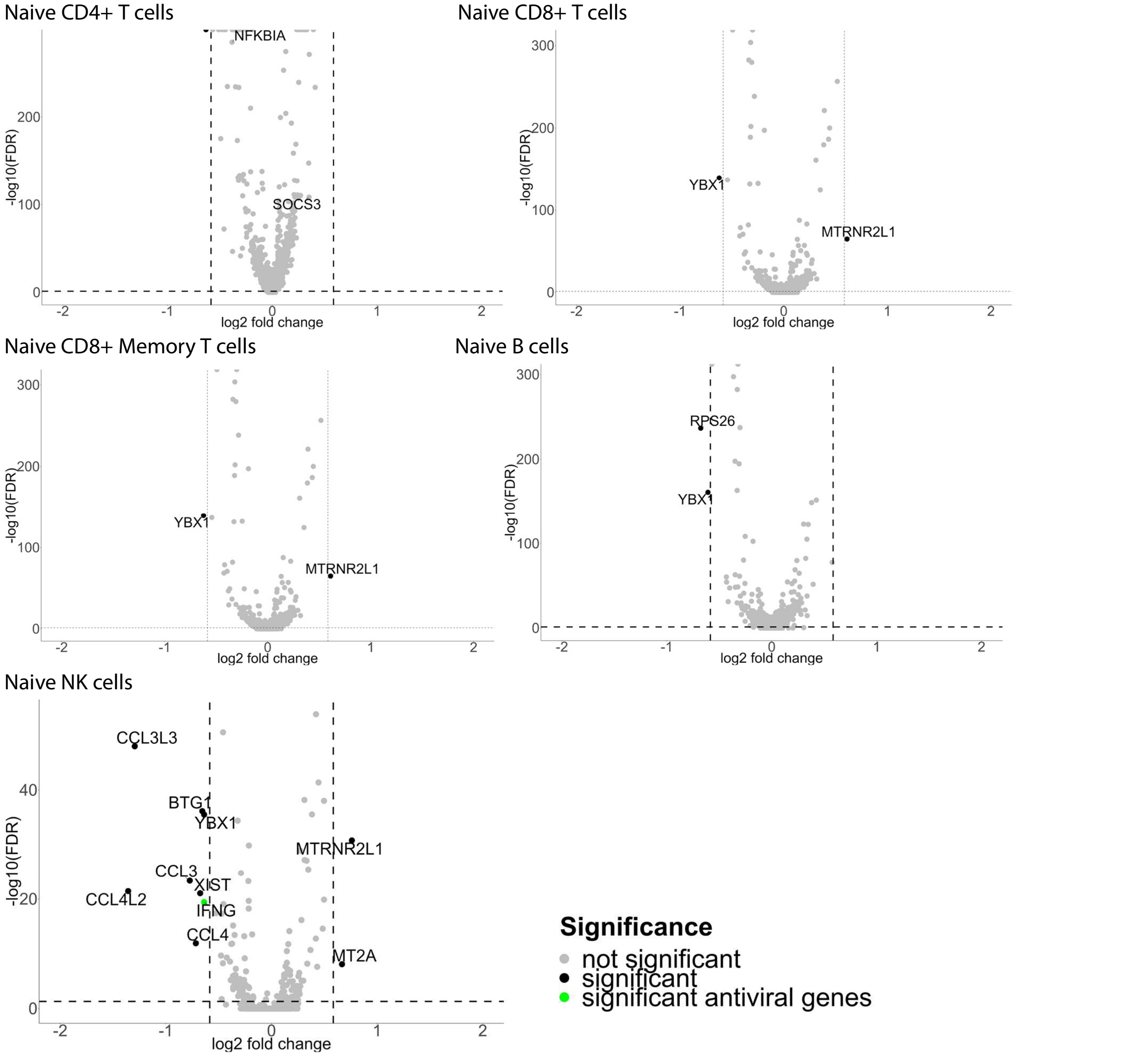

Supplementary Figure 4. Differential gene expression analysis of opioid-dependent and non-dependent control PBMCs across naive state cell types. We performed differential expression analysis within each cell type between control and opioid-dependent cells (see Methods). Volcano plot showing fold change of genes (log2 scale) for opioid-dependent cells compared to controls from Naive CD4+ T cells, Naive CD8+ T cells, Naive CD8+ memory T cells, Naive B cells, and Naive NK cells (x-axis) and significance of 0.05 (y-axis, -log10 scale). Significant genes shown in black, significant antiviral genes shown in green, and insignificant genes shown in grey. Source data listing genes and expression values are provided in Source Data file.

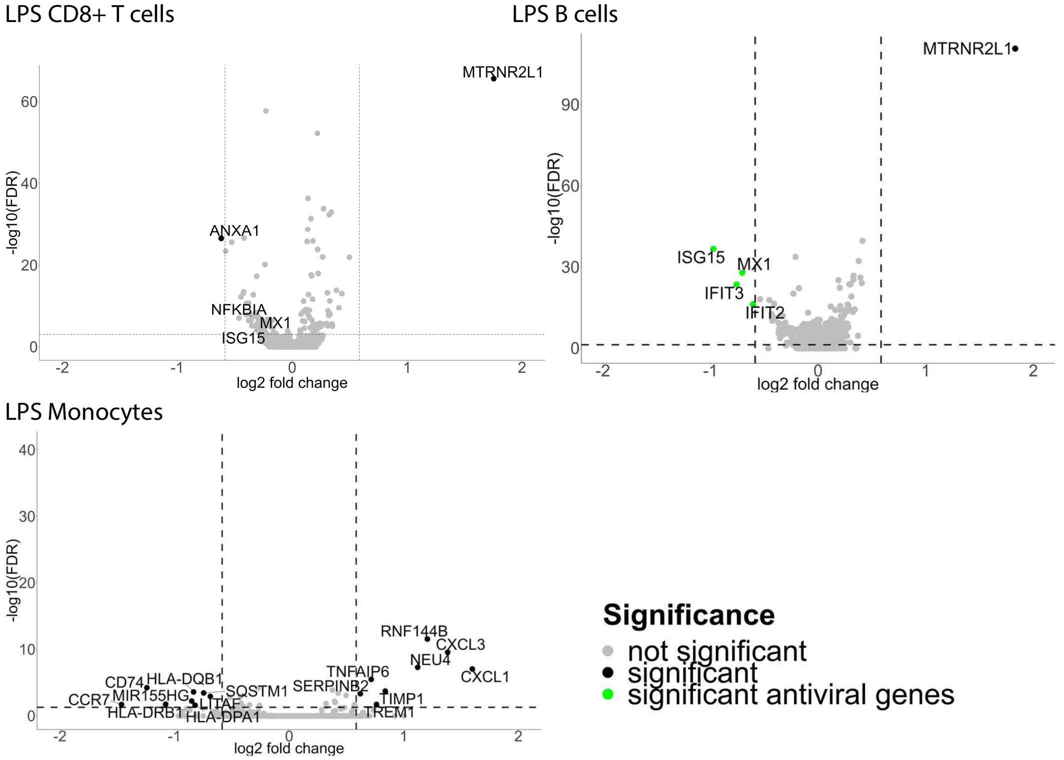

Supplementary Figure 5. Differential gene expression analysis of opioid-dependent and non-dependent control PBMCs across LPS treated cell types. We performed differential expression analysis within each cell type between control and opioid dependent cells (see Methods). Volcano plot showing fold change of genes (log2 scale) for opioid-dependent cells compared to controls from LPS treated populations: CD8+ T cells, B cells, Monocytes (x-axis) and significance of 0.05 (y-axis, -log10 scale). Significant genes shown in black, significant antiviral genes shown in green, and insignificant genes shown in grey. Source data listing genes and expression values are provided in Source Data file.

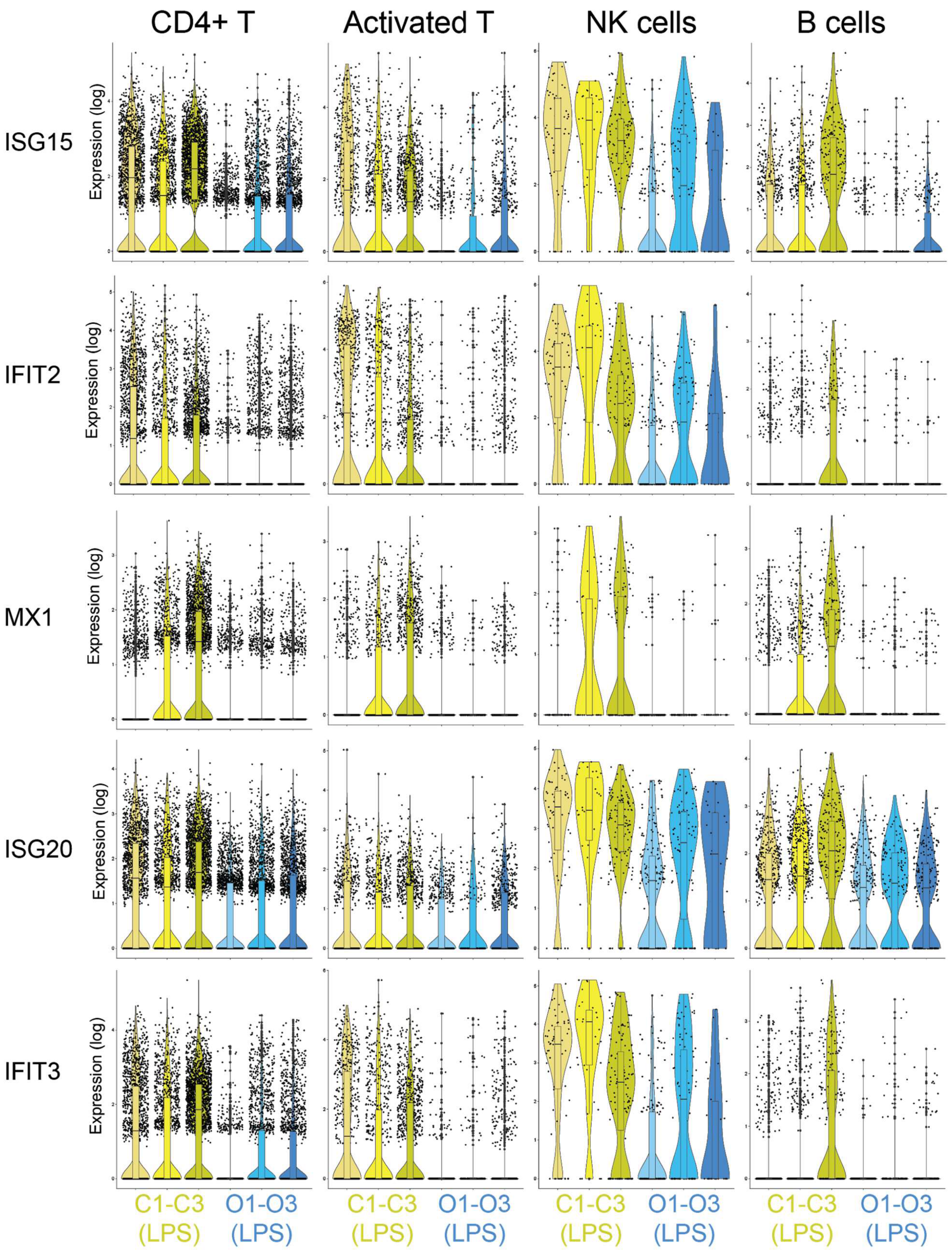

Supplementary Figures 6. Expression of key core antiviral genes in PBMCs of control and opioid-dependent individuals across LPS treated cell types and states. Violin plots of the log expression of key antiviral genes in each single cell, grouped by sample-of-origin for LPS treated populations: CD4+ T cells, activated T cells, NK cells, and B cells. Inset box plots show the median, lower and upper hinges that correspond to the first quartile (25th percentile) and third quartile (75th percentile), and the upper and lower whiskers extend from the smallest and largest hinges at most 1.5 times the interquartile range.

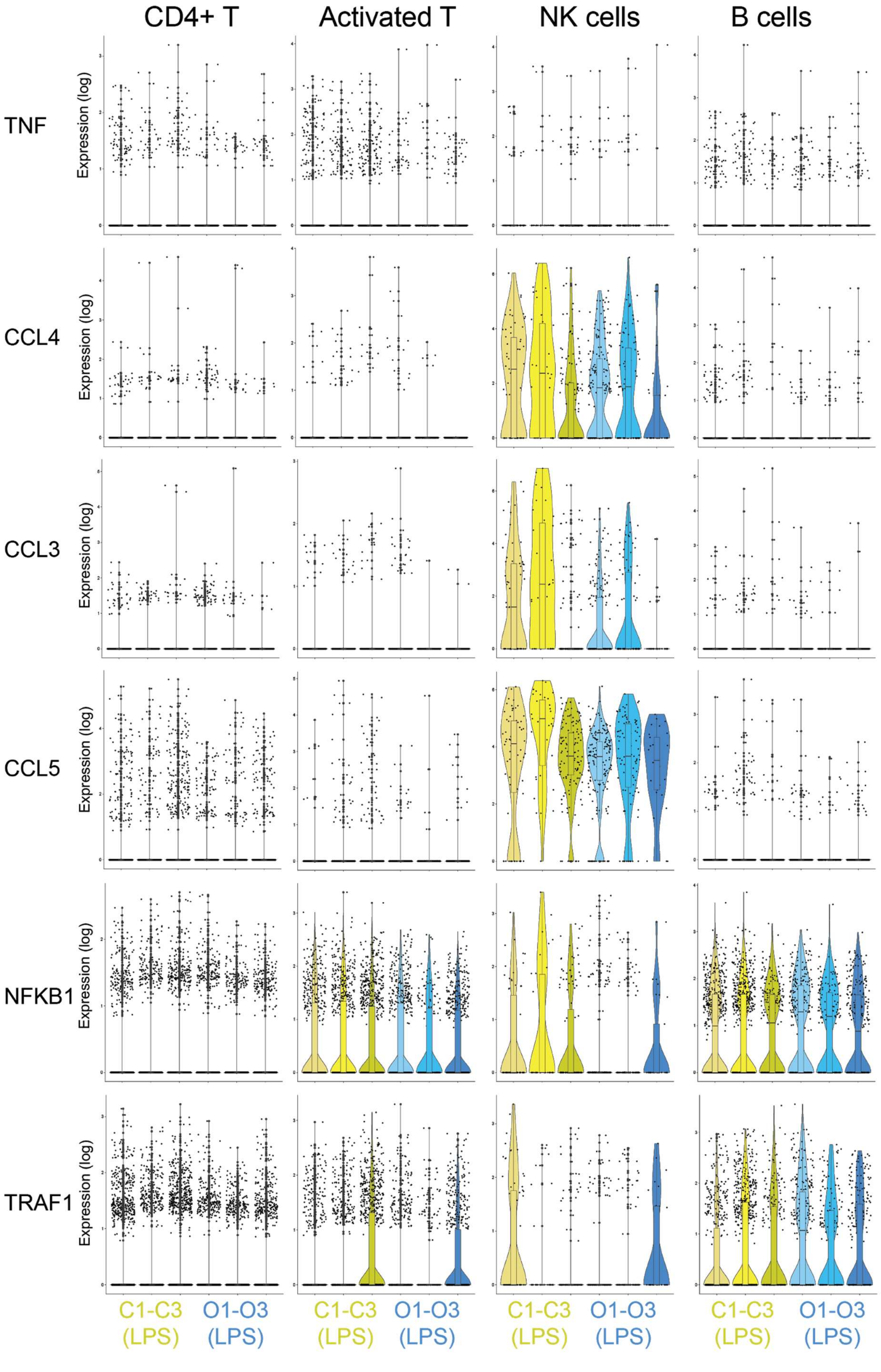

CD4+ T
Activated T
NK cells
B cells
TNF
CCL4
CCL3
CCL5
NFKB1
TRAF1
Expression (log)
C1-C3 (LPS)
O1-O3 (LPS)

Supplementary Figures 7. Expression of key inflammatory genes in PBMCs of control and opioid-dependent individuals across LPS treated cell types and states. Violin plots of the log expression of key inflammatory genes in each single cell, grouped by sample-of-origin for LPS treated populations: CD4+ T cells, activated T cells, NK cells, and B cells. Inset box plots show the median, lower and upper hinges that correspond to the first quartile (25th percentile) and third quartile (75th percentile), and the upper and lower whiskers extend from the smallest and largest hinges at most 1.5 times the interquartile range.

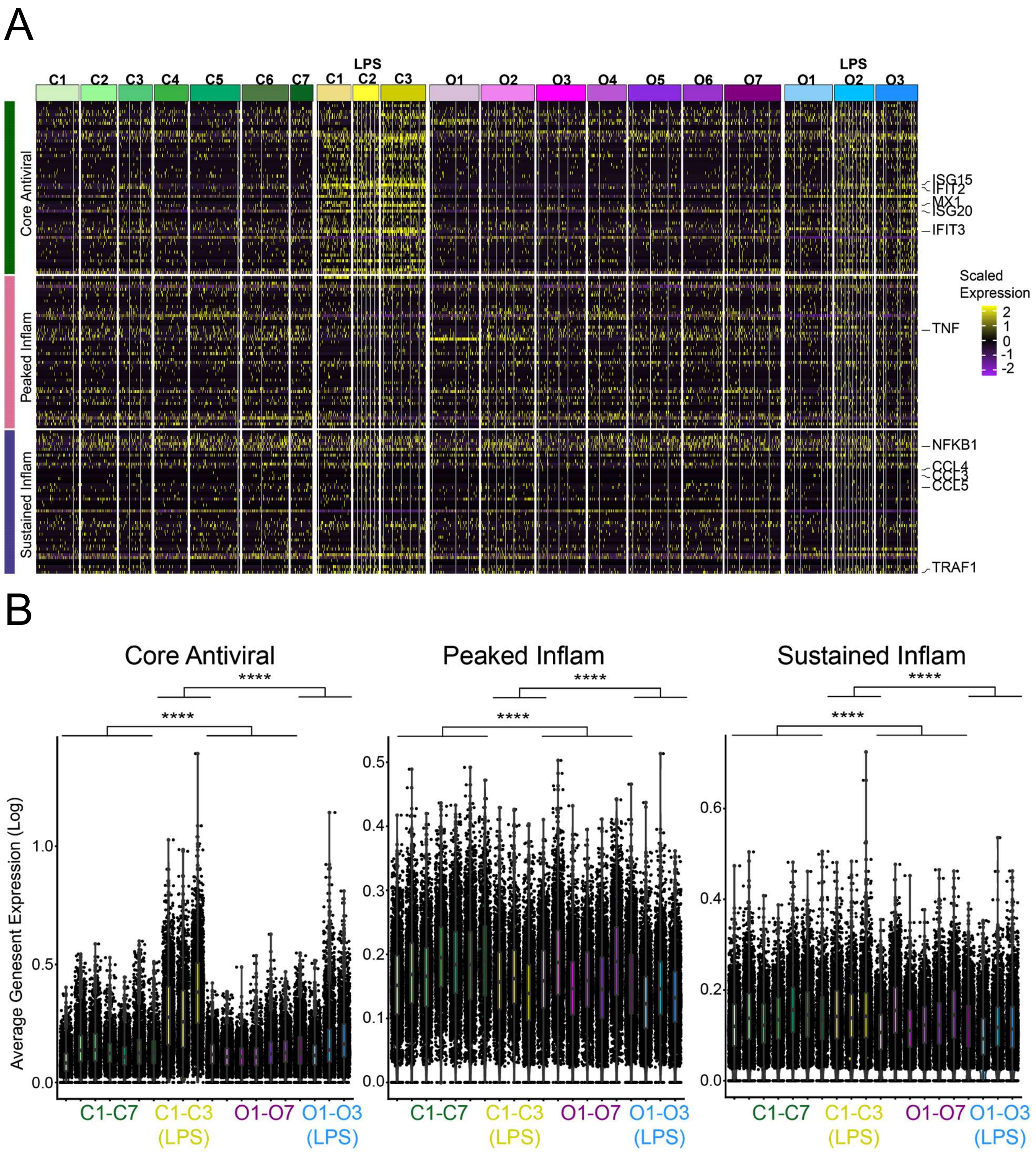

A
LPS
C1 C2 C3 C4 C5 C6 C7 C1 C2 C3 O1 O2 O3 O4 O5 O6 O7 O1 O2 O3
Core Antiviral
Peaked Inflam
Sustained Inflam
ISG15
IFIT2
MX1
ISG20
IFIT3
TNF
NFKB1
CCL4
CCL3
CCL5
TRAF1
Scaled Expression
2
1
0
-1
-2
B
Core Antiviral
Peaked Inflam
Sustained Inflam
****
Average Genesent Expression (Log)
C1-C7
C1-C3 (LPS)
O1-O7
O1-O3 (LPS)

Supplementary Figure 8. Single cell gene expression heatmap of antiviral and inflammatory gene modules in CD4+ T cells from LPS treated PBMCs of control and opioid-dependent individuals. a, Heatmap of scaled expression of core antiviral, peaked inflammatory, and sustained inflammatory genes (y-axis) for sample cells: naive state control samples (Naive Control), LPS treated control samples (LPS Control), naive state opioid-dependent samples (Naive Opioid), and LPS treated opioid-dependent samples (LPS Opioid). **b,** Average expression of all genes in each gene set (log expression) for each cell, grouped by samples:C1-C7 (naive control samples 1 - 7) (13275 cells), C1-C3 (LPS) (LPS treated control samples 1 - 3) (5211 cells), O1-O7 (naive opioid dependent samples 1-7) (16827 cells), O1-O3 (LPS) (LPS treated opioid dependent samples 1-3) (6378 cells).Inset box plots show the median, lower and upper hinges that correspond to the first quartile (25th percentile) and third quartile (75th percentile), and the upper and lower whiskers extend from the smallest and largest hinges at most 1.5 times the interquartile range. Two-tailed T-test with comparison tests between control and opioid-dependent groups for each geneset. For core antiviral gene set expression: comparison test between all naive control cells (C1-C7) and all naive opioid cells (O1-O7) ($p=7.7e-12$), as well as between all LPS treated control cells (C1-C3 (LPS)) and all LPS treated opioid dependent cells (O1-O3 (LPS)) ($p<2.22e-16$). For peaked inflammatory gene set expression: comparison test between all naive control cells (C1-C7) and all naive opioid cells (O1-O7) ($p<2.22e-16$), as well as between all LPS treated control cells (C1-C3 (LPS)) and all LPS treated opioid dependent cells (O1-O3 (LPS)) ($p<2.22e-16$). For sustained inflammatory gene set expression: comparison test between all naive control cells (C1-C7) and all naive opioid cells (O1-O7) ($p<2.22e-16$), as well as between all LPS treated control cells (C1-C3 (LPS)) and all LPS treated opioid dependent cells (O1-O3 (LPS)) ($p<2.22e-16$). $^{ns}p < 0.05$, $^{*}p < 0.05$, $^{**}p < 0.01$, $^{***}p < 0.001$, $^{****}p < 0.0001$.

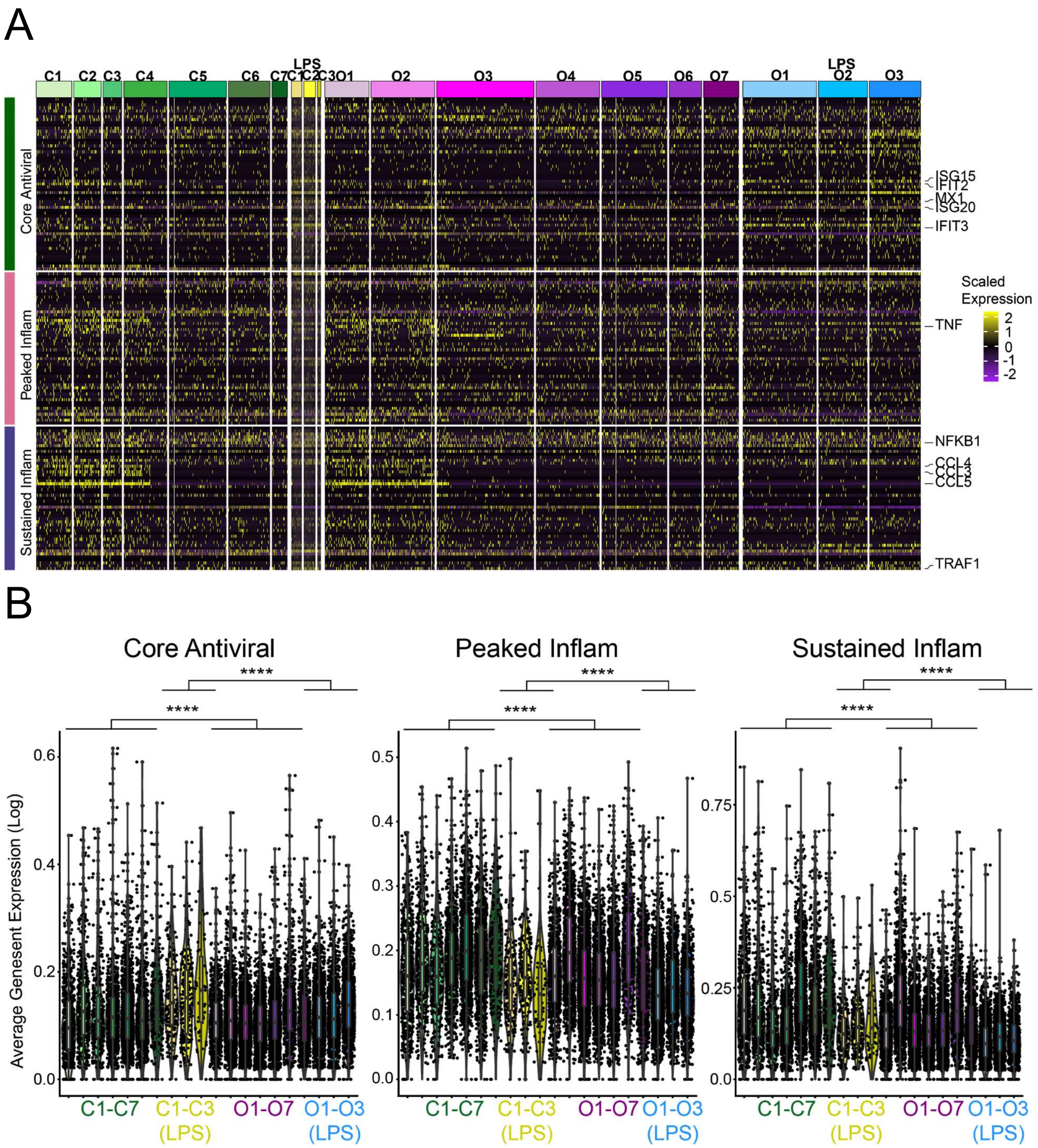

A
C1
C2
C3
C4
C5
C6
C7
LPS
C1
C2
C3
O1
O2
O3
O4
O5
O6
O7
O1
LPS
O2
O3
Core Antiviral
Peaked Inflam
Sustained Inflam
ISG15
IFIT2
MX1
ISG20
IFIT3
TNF
NFKB1
CCL4
CCL3
CCL5
TRAF1
Scaled Expression
2
1
0
-1
-2
B
Core Antiviral
Peaked Inflam
Sustained Inflam
****
Average Genesent Expression (Log)
C1-C7
C1-C3 (LPS)
O1-O7
O1-O3 (LPS)

Supplementary Figure 9. Single cell gene expression heatmap of antiviral and inflammatory gene modules in CD8+ T cells from LPS treated PBMCs of control and opioid-dependent individuals. a, Heatmap of scaled expression of core antiviral, peaked inflammatory, and sustained inflammatory gene modules (y-axis) for samples cells: naive state control samples (Naive Control), LPS treated control samples (LPS Control), naive state opioid-dependent samples (Naive Opioid), and LPS treated opioid-dependent samples (LPS Opioid). **b,** Average expression of all genes in each gene set (log expression) for each cell, grouped by samples: C1-C7 (naive control samples 1 - 7) (3750 cells), C1-C3 (LPS) (LPS treated-stimulated control samples 1 - 3) (439 cells), O1-O7 (naive opioid dependent samples 1-7) (6180 cells), O1-O3 (LPS) (LPS treated-stimulated opioid dependent samples 1-3) (2658 cells). Inset box plots show the median, lower and upper hinges that correspond to the first quartile (25th percentile) and third quartile (75th percentile), and the upper and lower whiskers extend from the smallest and largest hinges at most 1.5 times the interquartile range. Two-tailed T-test with comparison tests between control and opioid-dependent groups for each geneset. For core antiviral gene set expression: comparison test between all naive control cells (C1-C7) and all naive opioid cells (O1-O7) ($p=7.9e-15$), as well as between all LPS treated control cells (C1-C3 (LPS)) and all LPS treated opioid dependent cells (O1-O3 (LPS)) ($p=2.6e-13$). For peaked inflammatory gene set expression: comparison test between all naive control cells (C1-C7) and all naive opioid cells (O1-O7) ($p<2.22e-16$), as well as between all LPS treated control cells (C1-C3 (LPS)) and all LPS treated opioid dependent cells (O1-O3 (LPS)) ($p=3.3e-7$). For sustained inflammatory gene set expression: comparison test between all naive control cells (C1-C7) and all naive opioid cells (O1-O7) ($p<2.22e-16$), as well as between all LPS treated control cells (C1-C3 (LPS)) and all LPS treated opioid dependent cells (O1-O3 (LPS)) ($p<2.22e-16$). $^{ns}p < 0.05$, $^{*}p < 0.05$, $^{**}p < 0.01$, $^{***}p < 0.001$, $^{****}p < 0.0001$.

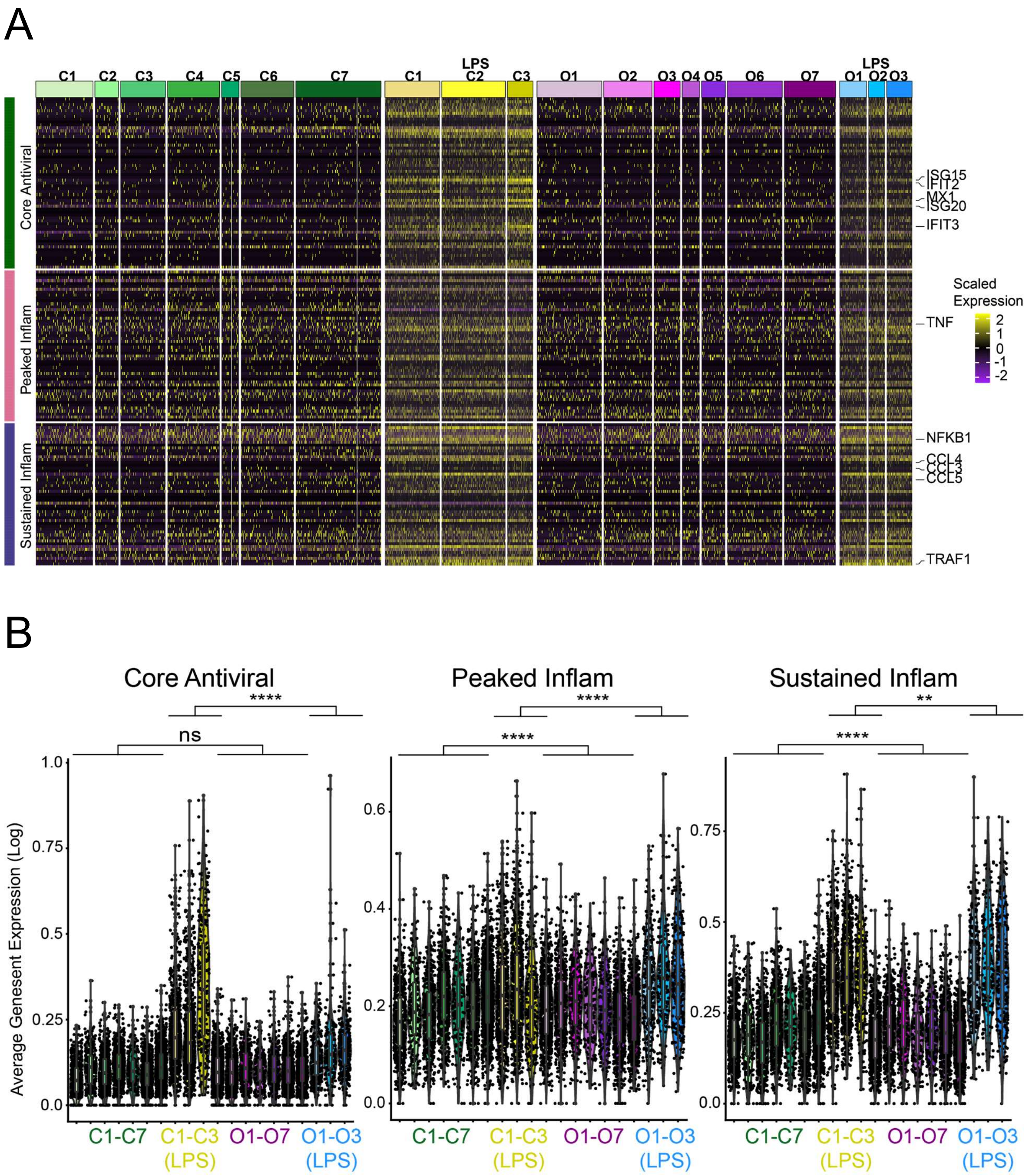
A
C1
C2
C3
C4
C5
C6
C7
LPS
C1
C2
C3
O1
O2
O3
O4
O5
O6
O7
LPS
O1
O2
O3
Core Antiviral
Peaked Inflam
Sustained Inflam
ISG15
IFIT2
MX1
ISG20
IFIT3
TNF
NFKB1
CCL4
CCL3
CCL5
TRAF1
Scaled
Expression
2
1
0
-1
-2
B
Core Antiviral
Peaked Inflam
Sustained Inflam
****
ns
****
****
**
****
Average Genesent Expression (Log)
1.0
0.75
0.5
0.25
0.0
0.6
0.4
0.2
0.0
0.75
0.5
0.25
0.0
C1-C7
C1-C3
(LPS)
O1-O7
O1-O3
(LPS)

Supplementary Figure 10. Single cell gene expression heatmap of antiviral and inflammatory gene modules in B cells from LPS treated PBMCs of control and opioid-dependent individuals. a, Heatmap of scaled expression of core antiviral, peaked inflammatory, and sustained inflammatory gene modules (y-axis) for samples cells: naive state control samples (Naive Control), LPS treated control samples (LPS Control), naive state opioid-dependent samples (Naive Opioid), and LPS treated opioid-dependent samples (LPS Opioid). **b,** Average expression of all genes in each gene set (log expression) for each cell, grouped by samples: C1-C7 (naive control samples 1 - 7) (3561 cells), C1-C3 (LPS) (LPS treated-stimulated control samples 1 - 3) (1527 cells), O1-O7 (naive opioid dependent samples 1-7) (3082 cells), O1-O3 (LPS) (LPS treated-stimulated opioid dependent samples 1-3) (747 cells). Inset box plots show the median, lower and upper hinges that correspond to the first quartile (25th percentile) and third quartile (75th percentile), and the upper and lower whiskers extend from the smallest and largest hinges at most 1.5 times the interquartile range. Two-tailed T-test with comparison tests Two-tailed T-test with comparison tests between control and opioid-dependent groups for each geneset. For core antiviral gene set expression: comparison test between all naive control cells (C1-C7) and all naive opioid cells (O1-O7) ($p=0.05$), as well as between all LPS treated control cells (C1-C3 (LPS)) and all LPS treated opioid dependent cells (O1-O3 (LPS)) ($p<2.2e-16$). For peaked inflammatory gene set expression: comparison test between all naive control cells (C1-C7) and all naive opioid cells (O1-O7) ($p=1.4e-6$), as well as between all LPS treated control cells (C1-C3 (LPS)) and all LPS treated opioid dependent cells (O1-O3 (LPS)) ($p=3.7e-6$). For sustained inflammatory gene set expression: comparison test between all naive control cells (C1-C7) and all naive opioid cells (O1-O7) ($p=1.4e-5$), as well as between all LPS treated control cells (C1-C3 (LPS)) and all LPS treated opioid dependent cells (O1-O3 (LPS)) ($p=0.0033$).$^{*}p < 0.05$, $^{**}p < 0.01$, $^{***}p < 0.001$, $^{****}p < 0.0001$.

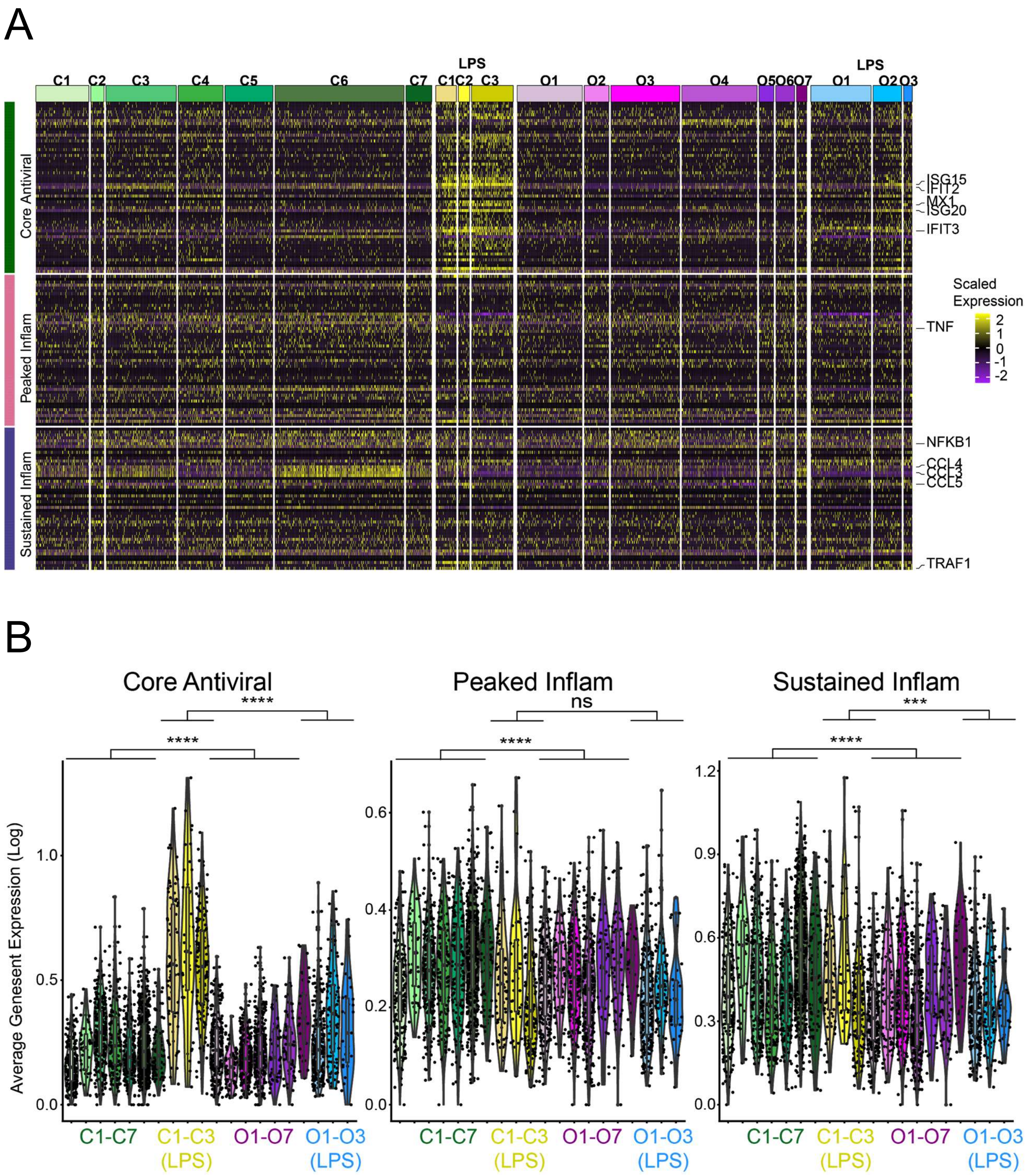
A
LPS
LPS
C1
C2
C3
C4
C5
C6
C7
C1
C2
C3
O1
O2
O3
O4
O5
O6
O7
O1
O2
O3
Core Antiviral
Peaked Inflam
Sustained Inflam
ISG15
IFIT2
MX1
ISG20
IFIT3
TNF
NFKB1
CCL4
CCL3
CCL5
TRAF1
Scaled Expression
2
1
0
-1
-2
B
Core Antiviral
Peaked Inflam
Sustained Inflam
****
****
ns
****
***
****
Average Genesent Expression (Log)
C1-C7
C1-C3 (LPS)
O1-O7
O1-O3 (LPS)

Supplementary Figure 11. Single cell gene expression heatmap of antiviral and inflammatory gene modules in NK cells from LPS treated PBMCs of control and opioid-dependent individuals. a, Heatmap of scaled expression of core antiviral, peaked inflammatory, and sustained inflammatory gene modules (y-axis) for samples cells: naive state control samples (Naive Control), LPS treated control samples (LPS Control), naive state opioid-dependent samples (Naive Opioid), and LPS treated opioid-dependent samples (LPS Opioid). **b,** Average expression of all genes in each gene set (log expression) for each cell, grouped by samples: C1-C7 (naive control samples 1 - 7) (1371 cells), C1-C3 (LPS) (LPS treated-stimulated control samples 1 - 3) (268 cells), O1-O7 (naive opioid dependent samples 1-7) (1003 cells), O1-O3 (LPS) (LPS treated-stimulated opioid dependent samples 1-3) (351 cells). Inset box plots show the median, lower and upper hinges that correspond to the first quartile (25th percentile) and third quartile (75th percentile), and the upper and lower whiskers extend from the smallest and largest hinges at most 1.5 times the interquartile range. Two-tailed T-test with comparison tests between control and opioid-dependent groups for each geneset. For core antiviral gene set expression: comparison test between all naive control cells (C1-C7) and all naive opioid cells (O1-O7) (p=6e-05), as well as between all LPS treated control cells (C1-C3 (LPS)) and all LPS treated opioid dependent cells (O1-O3 (LPS)) (p<2.22e-16). For peaked inflammatory gene set expression: comparison test between all naive control cells (C1-C7) and all naive opioid cells (O1-O7) (p=9.3e-13), as well as between all LPS treated control cells (C1-C3 (LPS)) and all LPS treated opioid dependent cells (O1-O3 (LPS)) (p=0.44). For sustained inflammatory gene set expression: comparison test between all naive control cells (C1-C7) and all naive opioid cells (O1-O7) (p<2.22e-16), as well as between all LPS treated control cells (C1-C3 (LPS)) and all LPS treated opioid dependent cells (O1-O3 (LPS)) (p=0.00054). $^{ns}p < 0.05$, *p < 0.05, **p < 0.01, ***p < 0.001, ****p < 0.0001.

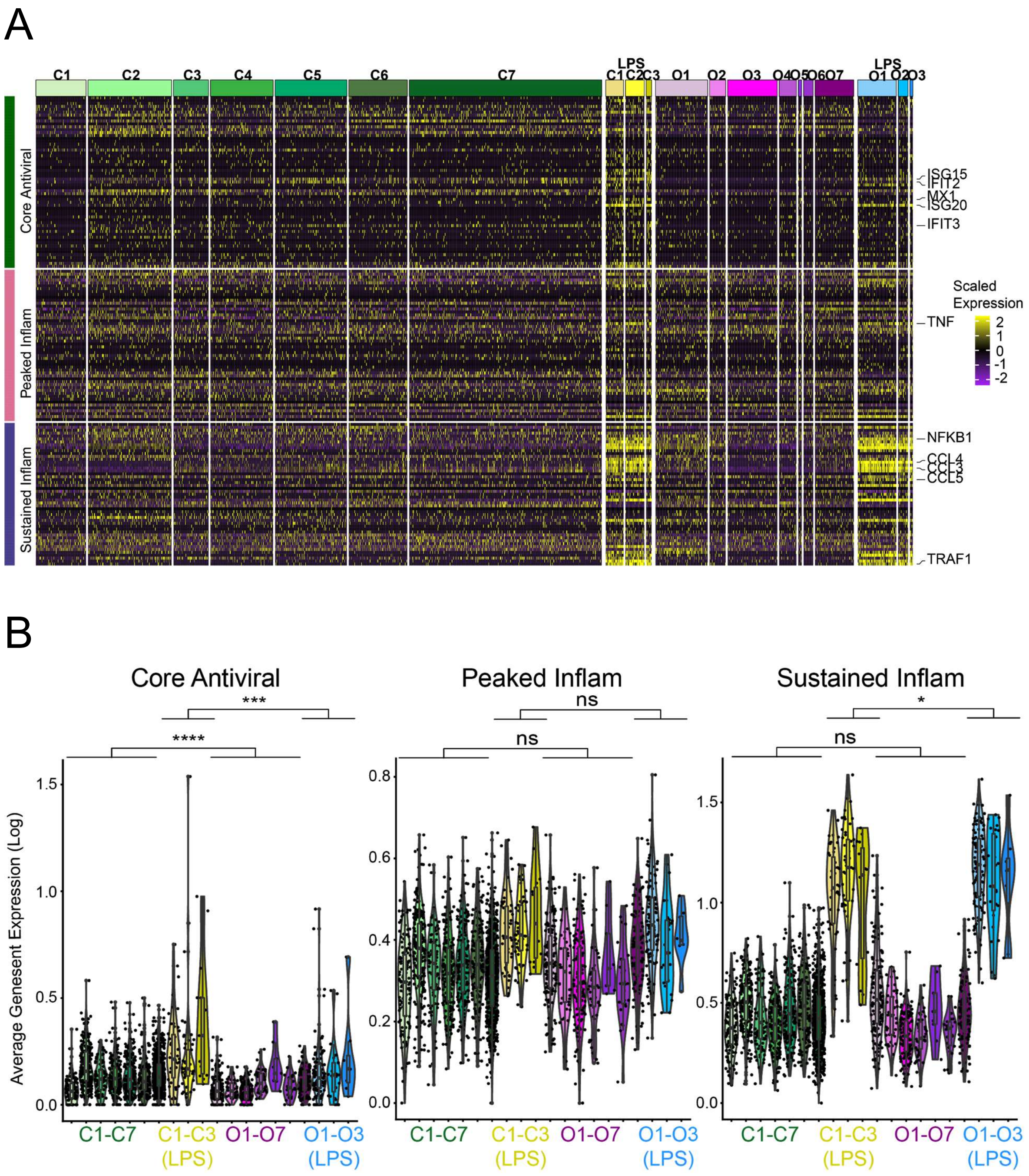

A
C1
C2
C3
C4
C5
C6
C7
LPS
C1 C2 C3
O1
O2
O3
O4 O5 O6 O7
LPS
O1 O2 O3
Core Antiviral
Peaked Inflam
Sustained Inflam
ISG15
IFIT2
MX1
ISG20
IFIT3
TNF
NFKB1
CCL4
CCL3
CCL5
TRAF1
Scaled Expression
2
1
0
-1
-2
B
Core Antiviral
Peaked Inflam
Sustained Inflam
Average Genesent Expression (Log)
***
****
ns
ns
ns
*
C1-C7
C1-C3 (LPS)
O1-O7
O1-O3 (LPS)

Supplementary Figure 12. Single cell gene expression heatmap of antiviral and inflammatory gene modules in Monocytes from LPS treated PBMCs of control and opioid-dependent individuals. a, Heatmap of scaled expression of core antiviral, peaked inflammatory, and sustained inflammatory gene modules (y-axis) for samples cells: naive state control samples (Naive Control), LPS treated control samples (LPS Control), naive state opioid-dependent samples (Naive Opioid), and LPS treated opioid-dependent samples (LPS Opioid). **b,** Average expression of all genes in each gene set (log expression) for each cell, grouped by samples: C1-C7 (naive control samples 1 - 7) (1459 cells), C1-C3 (LPS) (LPS treated-stimulated control samples 1 - 3) (118 cells), O1-O7 (naive opioid dependent samples 1-7) (511 cells), O1-O3 (LPS) (LPS treated-stimulated opioid dependent samples 1-3) (142 cells). Inset box plots show the median, lower and upper hinges that correspond to the first quartile (25th percentile) and third quartile (75th percentile), and the upper and lower whiskers extend from the smallest and largest hinges at most 1.5 times the interquartile range. Two-tailed T-test with comparison tests between control and opioid-dependent groups for each geneset. For core antiviral gene set expression: comparison test between all naive control cells (C1-C7) and all naive opioid cells (O1-O7) ($p<2.22e-16$), as well as between all LPS treated control cells (C1-C3 (LPS)) and all LPS treated opioid dependent cells (O1-O3 (LPS)) ($p=0.00015$). For peaked inflammatory gene set expression: comparison test between all naive control cells (C1-C7) and all naive opioid cells (O1-O7) ($p=0.44$), as well as between all LPS treated control cells (C1-C3 (LPS)) and all LPS treated opioid dependent cells (O1-O3 (LPS)) ($p=0.64$). For sustained inflammatory gene set expression: comparison test between all naive control cells (C1-C7) and all naive opioid cells (O1-O7) ($p=0.15$), as well as between all LPS treated control cells (C1-C3 (LPS)) and all LPS treated opioid dependent cells (O1-O3 (LPS)) ($p=0.021$). $^{ns}p < 0.05$, $^{*}p < 0.05$, $^{**}p < 0.01$, $^{***}p < 0.001$, $^{****}p < 0.0001$.

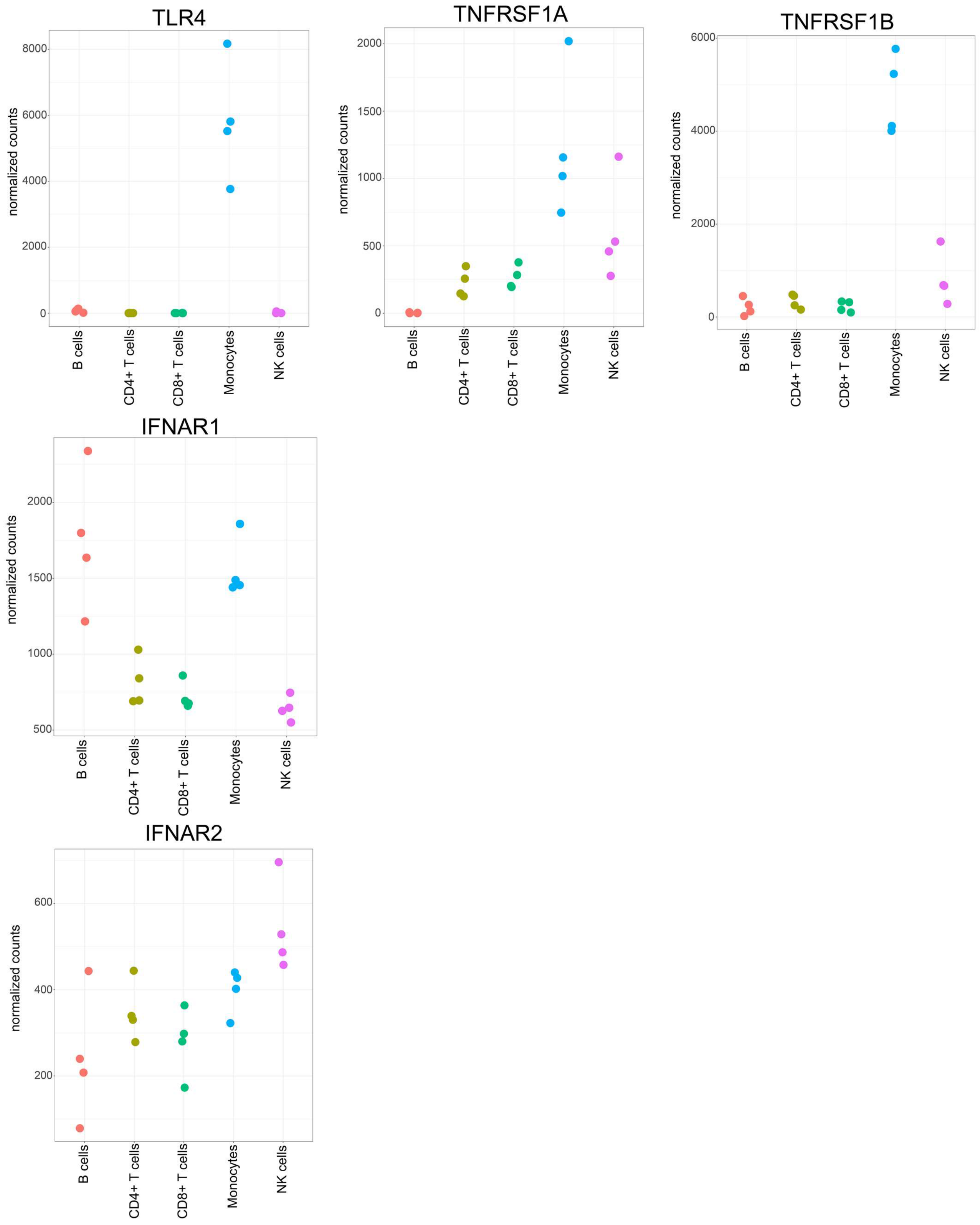

Supplementary Figure 13. TLR4, TNF receptor and IFN receptor gene expression in peripheral blood immune cell populations from bulk RNA-seq. Bulk RNA-seq data was analyzed from Corces et al. 2016[28] was reanalyzed. Normalized expression of opioid receptor genes TLR4, TNFRSF1A, TNFRSF1B, IFNAR1, IFNAR2 in healthy control donors across immune cell types: B cells, CD4+ T cells, CD8+ T cells, Monocytes, NK cells. Source data listing genes and expression values are provided in Source Data file.

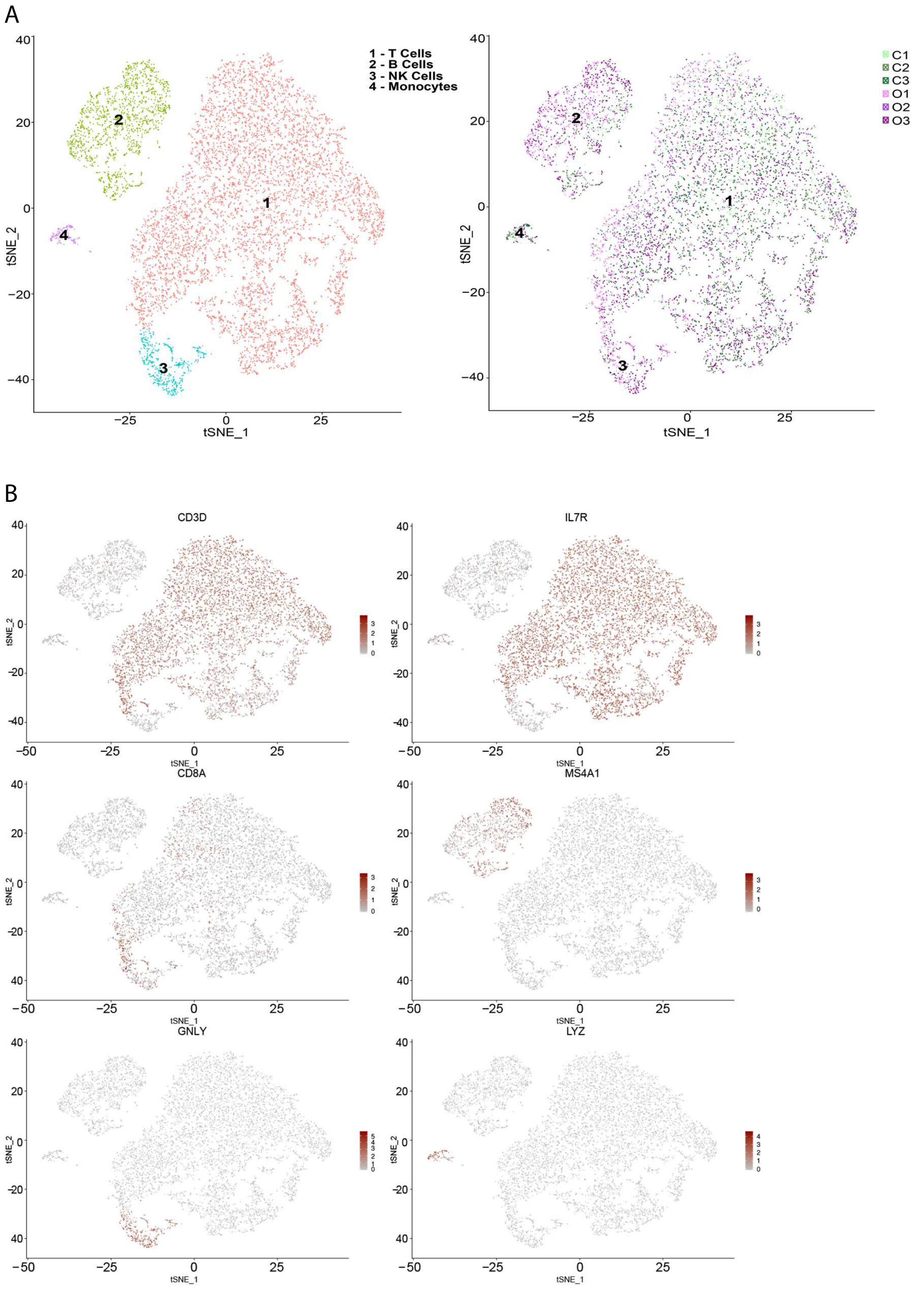

Supplementary Figure 14. Cell type identification of IFNβ-treated PBMCs from control and opioid-dependent individuals. a, t-SNE projection of unsupervised clustering of 9278 HTO cells. We identified cell type populations (left): T cells (7163 cells), B cells (1481cells), NK cells (473 cells), Monocytes (161 cells). We also colored cells by the 6 HTO samples: 3 control samples (C1-C3) and 3 opioid-dependent samples (O1-O3) (right). b, t-SNE projection of canonical gene marker expression across all subpopulations: CD4+ T cells (CD3D, IL7R), CD8+ T cells (CD3D, CD8B), B cells (MS4A1), NK cells (GNLY), and Monocytes (LYZ).

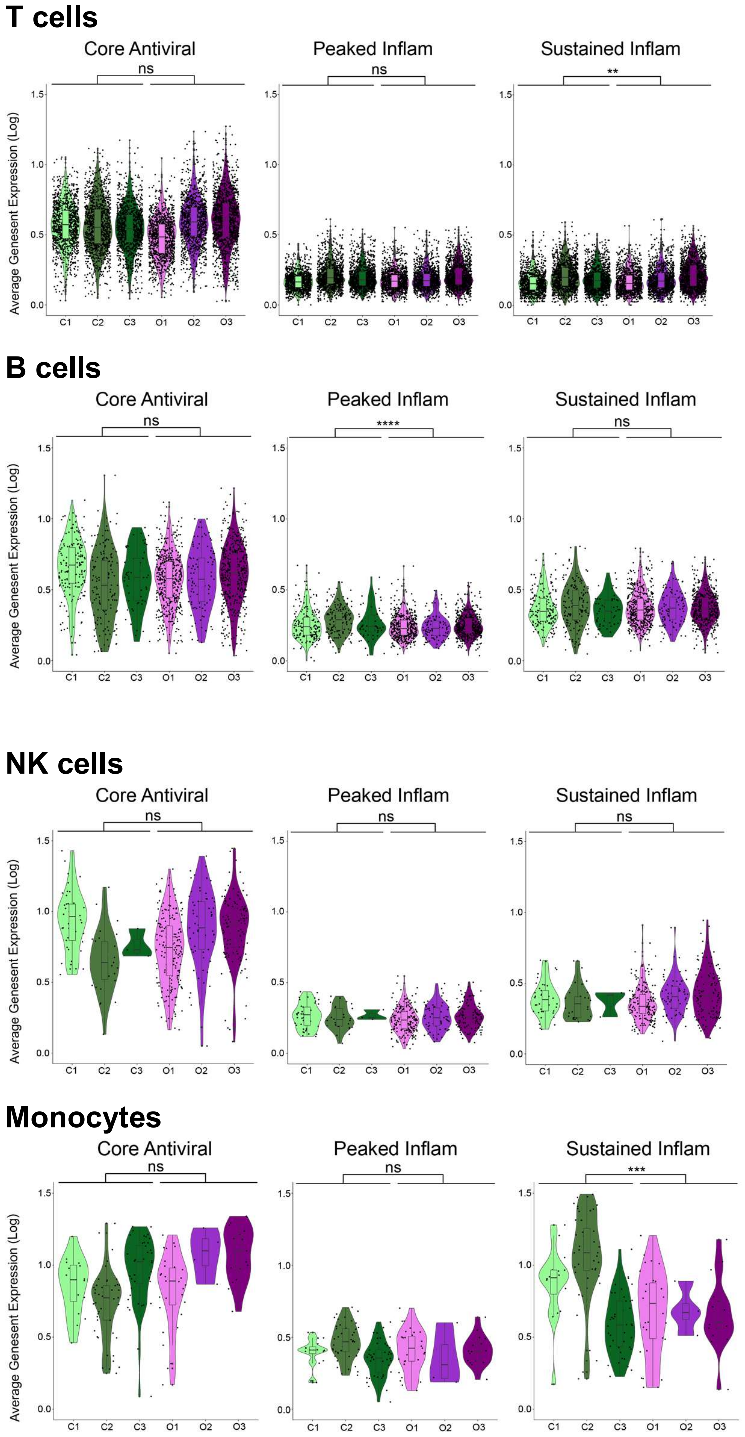
T cells
Core Antiviral
ns
Peaked Inflam
ns
Sustained Inflam
**
Average Genesent Expression (Log)
0.0
0.5
1.0
1.5
C1
C2
C3
O1
O2
O3
B cells
Core Antiviral
ns
Peaked Inflam
****
Sustained Inflam
ns
NK cells
Core Antiviral
ns
Peaked Inflam
ns
Sustained Inflam
ns
Monocytes
Core Antiviral
ns
Peaked Inflam
ns
Sustained Inflam
***

Supplementary Figure 15. Single cell gene expression of antiviral and inflammatory gene modules in IFNβ-treated PBMCs. Average expression of all genes in each gene set (log expression) for each cell, grouped by sample-of-origin in cell types: T cells (C1-C3: 3494 cells and O1-O3: 3669 cells), B cells (C1-C3:402 cells and O1-O3: 1079 cells), NK cells(C1-C3: 78 cells and O1-O3: 395 cells), and monocytes (C1-C3: 109 cells and O1-O3: 52 cells). Inset box plots show the median, lower and upper hinges that correspond to the first quartile (25th percentile) and third quartile (75th percentile), and the upper and lower whiskers extend from the smallest and largest hinges at most 1.5 times the interquartile range. For T cells, two-tailed T-test with comparison tests between control and opioid-dependent groups for each geneset: core antiviral (p=0.59), peaked inflammation (p=0.5), sustained inflammation (p=0.0024). For B cells, two-tailed T-test with comparison tests between control and opioid-dependent groups for each geneset: core antiviral (p=0.61), peaked inflammation (p=3.7e-06), sustained inflammation (p=0.71). For NK cells, two-tailed T-test with comparison tests between control and opioid-dependent groups for each geneset: core antiviral (p=0.77), peaked inflammation (p=0.42), sustained inflammation (p=0.33). For monocytes, two-tailed T-test with comparison tests between control and opioid-dependent groups for each geneset: core antiviral (p=0.061), peaked inflammation (p=0.81), sustained inflammation (p=0.00046).. $^{ns}p > 0.05$, $^{*}p < 0.05$, $^{**}p < 0.01$, $^{***}p < 0.001$, $^{****}p < 0.0001$.

A

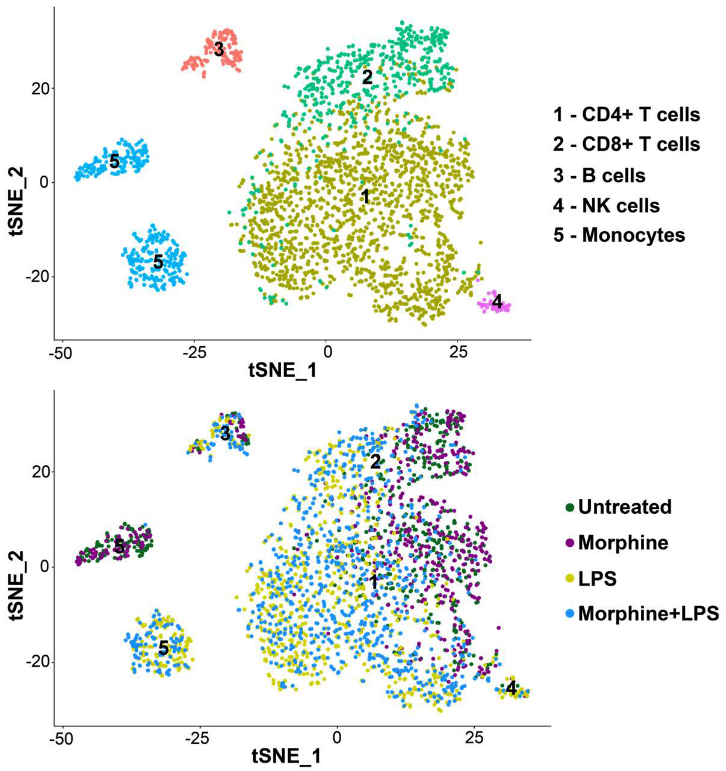

B

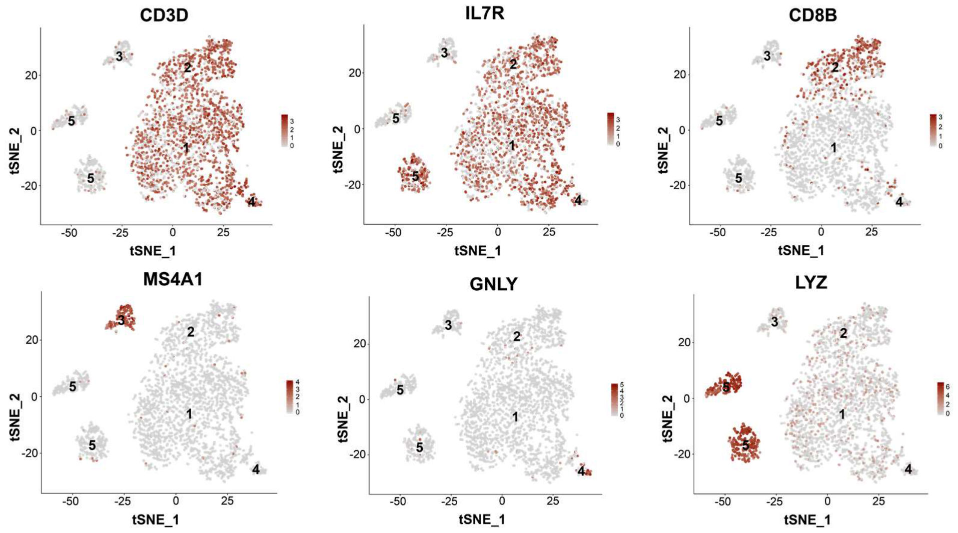

Supplementary Figure 16. Cell type identification of in vitro morphine-treated PBMCs. a, t-SNE projection of unsupervised clustering of 2,946 HTO cells. We identified cell type populations (top): CD4+ T cells (1,774 cells), CD8+ T cells (546 cells), B cells (152 cells), NK cells (58 cells), Monocytes (416 cells). We also colored cells by the 4 HTO samples in naive and LPS treated states (bottom). **b,** t-SNE projection of canonical gene marker expression across all subpopulations: CD4+ T cells (CD3D, IL7R), CD8+ T cells (CD3D, CD8B), B cells (MS4A1), NK cells (GNLY), and Monocytes (LYZ).

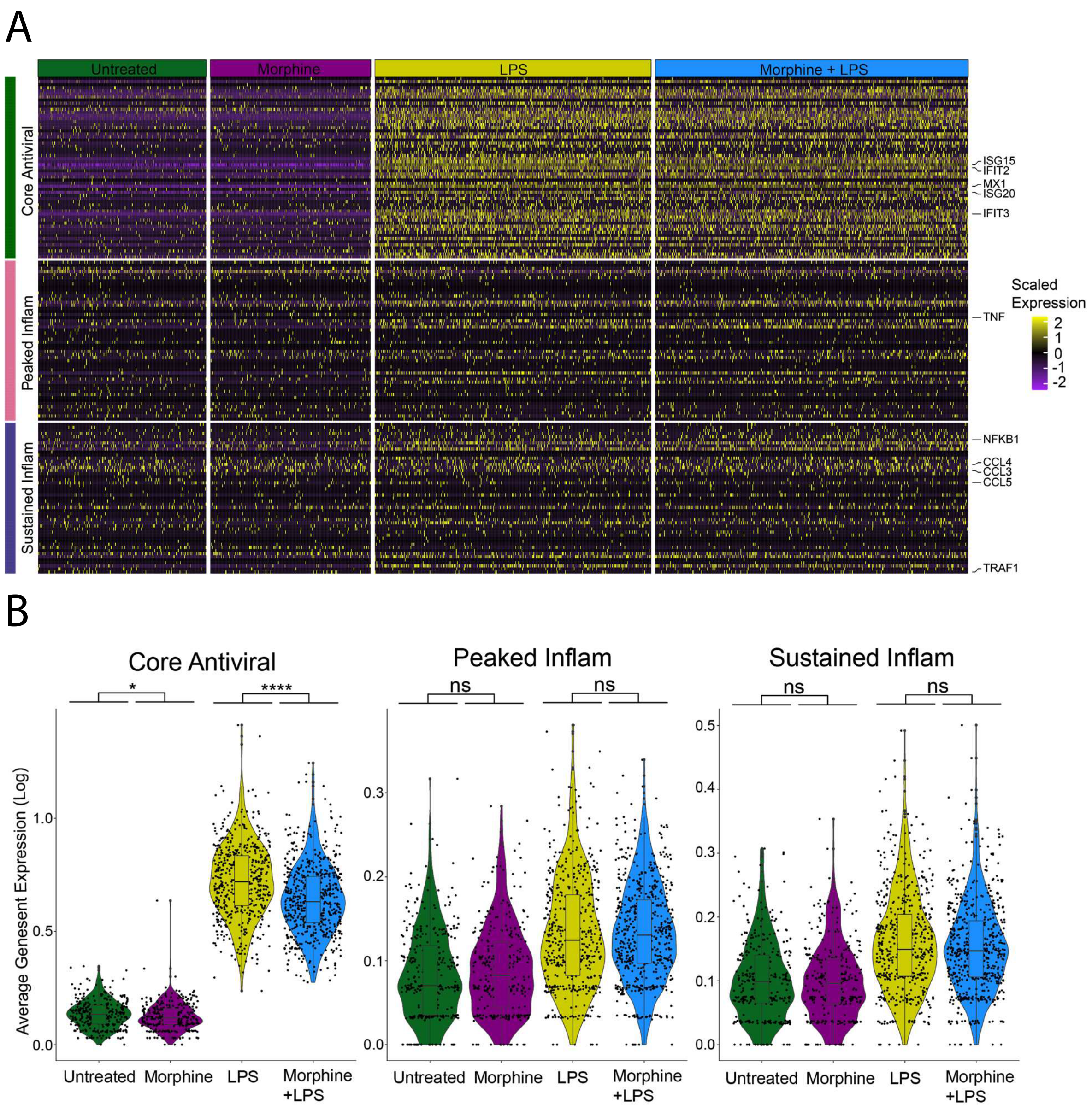

Supplementary Figure 17. Single cell gene expression heatmap of antiviral and inflammatory gene modules in in vitro treated CD4+ T cells. a, Heatmap of scaled expression of core antiviral, peaked inflammatory, and sustained inflammatory gene modules observed across all naive state samples and LPS treated samples . **b,** Average expression of all genes in each gene set (log expression) for each cell, grouped naive state samples (Untreated (325 cells), Morphine (310 cells)) and LPS treated samples (LPS (534 cells), Morphine+LPS (605 cells)). Inset box plots show the median, lower and upper hinges that correspond to the first quartile (25th percentile) and third quartile (75th percentile), and the upper and lower whiskers extend from the smallest and largest hinges at most 1.5 times the interquartile range. Two-tailed T-test with comparison tests between control and morphine treated groups for each gene set. For core antiviral gene set expression: comparison tests between Untreated and Morphine ($p=0.028$), as well as LPS and Morphine+LPS ($p<2.22e-16$). For peaked inflammatory gene set expression: comparison tests between Untreated and Morphine ($p=0.23$), as well as LPS and Morphine+LPS ($p=0.91$). For sustained inflammatory gene set expression: comparison tests between Untreated and Morphine ($p=0.56$), as well as LPS and Morphine+LPS ($p=0.16$). $^{ns}p > 0.05$, $^{*}p < 0.05$, $^{**}p < 0.01$, $^{***}p < 0.001$, $^{****}p < 0.0001$.

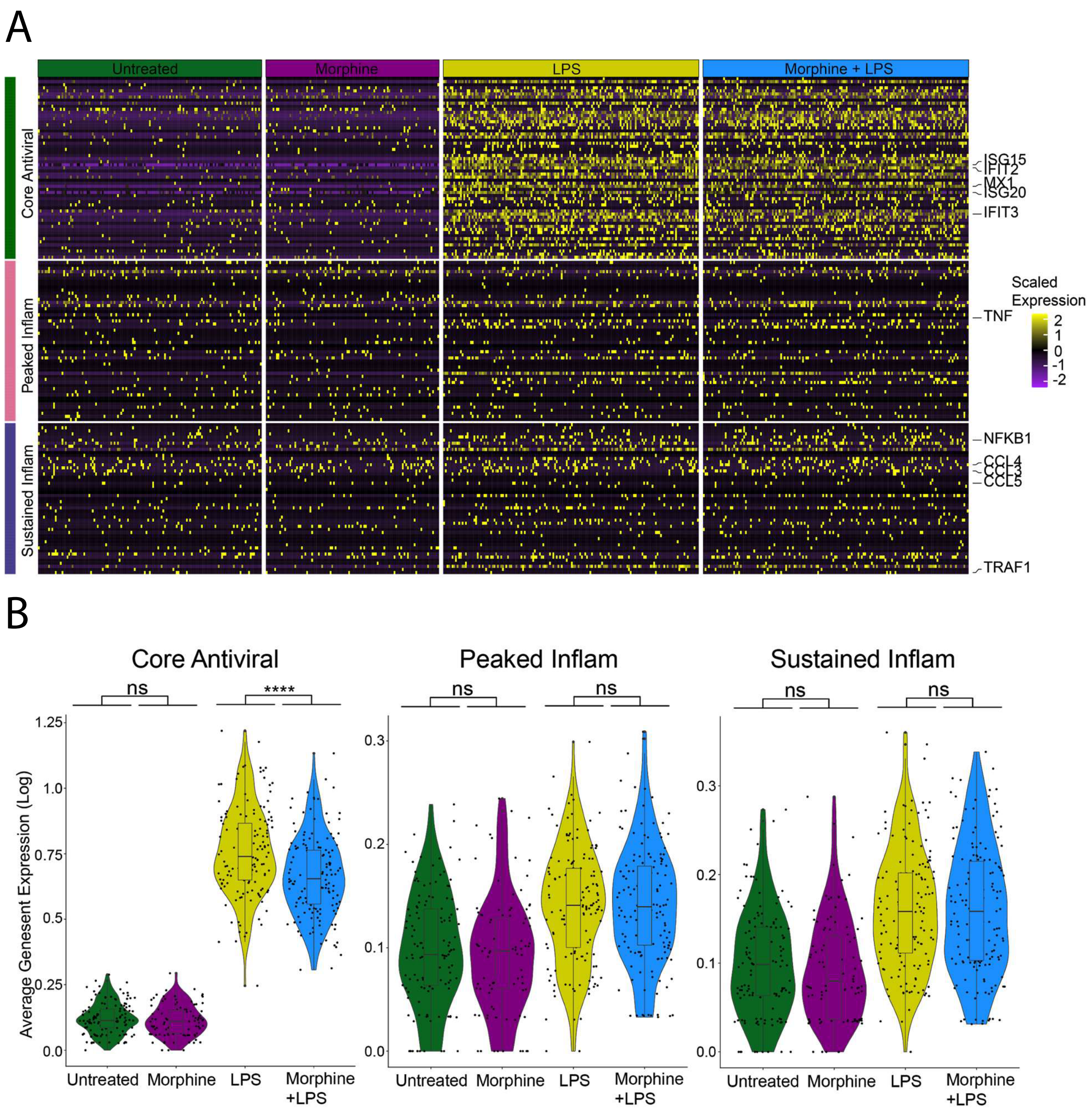

Supplementary Figure 18. Single cell gene expression heatmap of antiviral and inflammatory gene modules in in vitro treated CD8+ T cells. a, Heatmap of scaled expression of core antiviral, peaked inflammatory, and sustained inflammatory gene modules observed across all naive state samples and LPS treated samples . **b,** Average expression of all genes in each gene set (log expression) for each cell, grouped naive state samples (Untreated (133 cells), Morphine (103 cells)) and LPS treated samples (LPS (152 cells), Morphine+LPS (158 cells)). Inset box plots show the median, lower and upper hinges that correspond to the first quartile (25th percentile) and third quartile (75th percentile), and the upper and lower whiskers extend from the smallest and largest hinges at most 1.5 times the interquartile range. Two-tailed T-test with comparison tests between control and morphine treated groups for each gene set.: For core antiviral gene set expression: comparison tests between Untreated and Morphine ($p=0.53$), as well as LPS and Morphine+LPS ($p=6.3e-07$). For peaked inflammatory gene set expression: comparison tests between Untreated and Morphine ($p=0.5$), as well as LPS and Morphine+LPS ($p=0.91$). For sustained inflammatory gene set expression: comparison tests between Untreated and Morphine ($p=0.26$), as well as LPS and Morphine+LPS ($p=0.85$). $^{ns}p > 0.05$, $*p < 0.05$, $**p < 0.01$, $***p < 0.001$, $****p < 0.0001$.

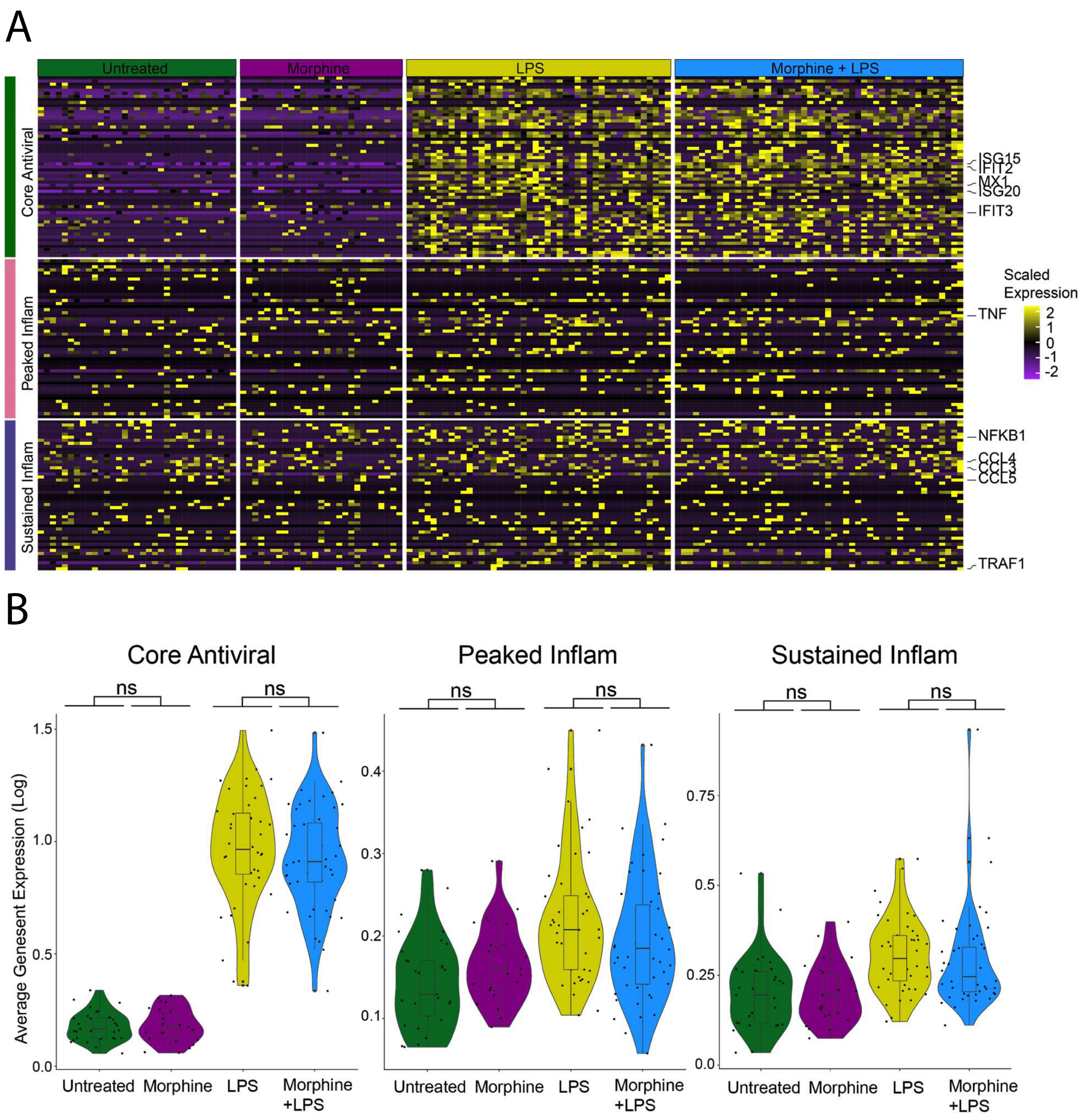

Supplementary Figure 19. Single cell gene expression of antiviral and inflammatory gene modules in in vitro treated B cells. a, Heatmap of scaled expression of core antiviral, peaked inflammatory, and sustained inflammatory gene modules observed across all naive state samples and LPS treated samples . **b,** Average expression of all genes in each gene set (log expression) for each cell, grouped naive state samples (Untreated (33 cells), Morphine (27 cells)) and LPS treated samples (LPS (44 cells), Morphine+LPS (48 cells)). Inset box plots show the median, lower and upper hinges that correspond to the first quartile (25th percentile) and third quartile (75th percentile), and the upper and lower whiskers extend from the smallest and largest hinges at most 1.5 times the interquartile range. Two-tailed T-test with comparison tests between control and morphine treated groupsfor each gene set.: For core antiviral gene set expression: comparison tests between Untreated and Morphine (p=0.9), as well as LPS and Morphine+LPS (p=0.38). For peaked inflammatory gene set expression: comparison tests between Untreated and Morphine (p=0.095), as well as LPS and Morphine+LPS (p=0.14). For sustained inflammatory gene set expression: comparison tests between Untreated and Morphine (p=0.88), as well as LPS and Morphine+LPS (p=0.55). $^{ns}p > 0.05$, $^{*}p < 0.05$, $^{**}p < 0.01$, $^{***}p < 0.001$, $^{****}p < 0.0001$.

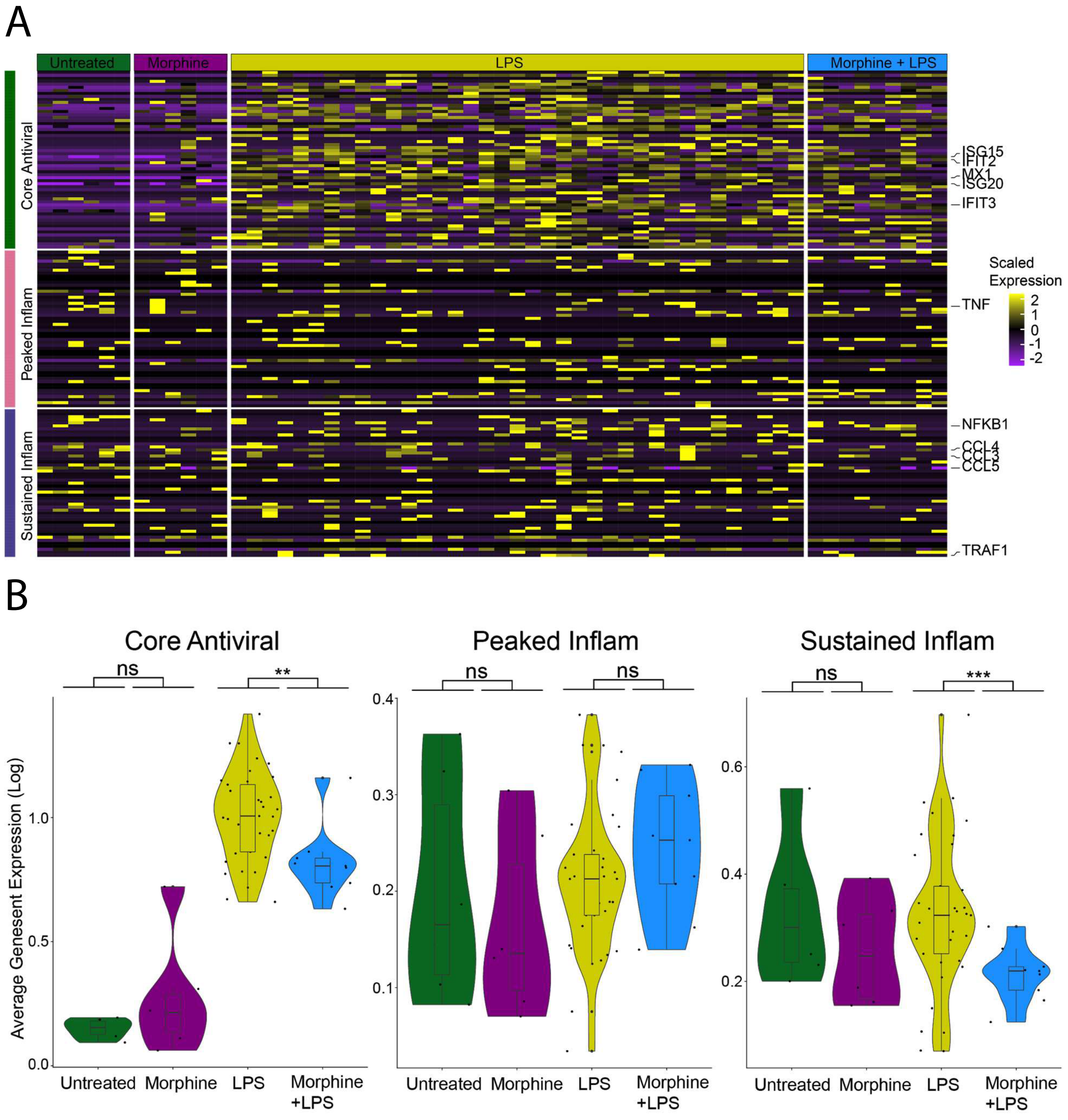

Supplementary Figure 20. Single cell gene expression of antiviral and inflammatory gene modules in in vitro treated NK cells. a, Heatmap of scaled expression of core antiviral, peaked inflammatory, and sustained inflammatory gene modules observed across all naive state samples and LPS treated samples. **b,** Average expression of all genes in each gene set (log expression) for each cell, grouped naive state samples (Untreated (6 cells), Morphine (6 cells)) and LPS treated samples (LPS (37 cells), Morphine+LPS (9 cells)). Inset box plots show the median, lower and upper hinges that correspond to the first quartile (25th percentile) and third quartile (75th percentile), and the upper and lower whiskers extend from the smallest and largest hinges at most 1.5 times the interquartile range. Two-tailed T-test with comparison tests between control and morphine treated groupsfor each gene set.: For core antiviral gene set expression: comparison tests between Untreated and Morphine ($p=0.27$), as well as LPS and Morphine+LPS ($p=0.0053$). For peaked inflammatory gene set expression: comparison tests between Untreated and Morphine ($p=0.57$), as well as LPS and Morphine+LPS ($p=0.23$). For sustained inflammatory gene set expression: comparison tests between Untreated and Morphine ($p=0.31$), as well as LPS and Morphine+LPS ($p=0.00039$). $^{*}p < 0.05$, $^{**}p < 0.01$, $^{***}p < 0.001$, $^{****}p < 0.0001$.

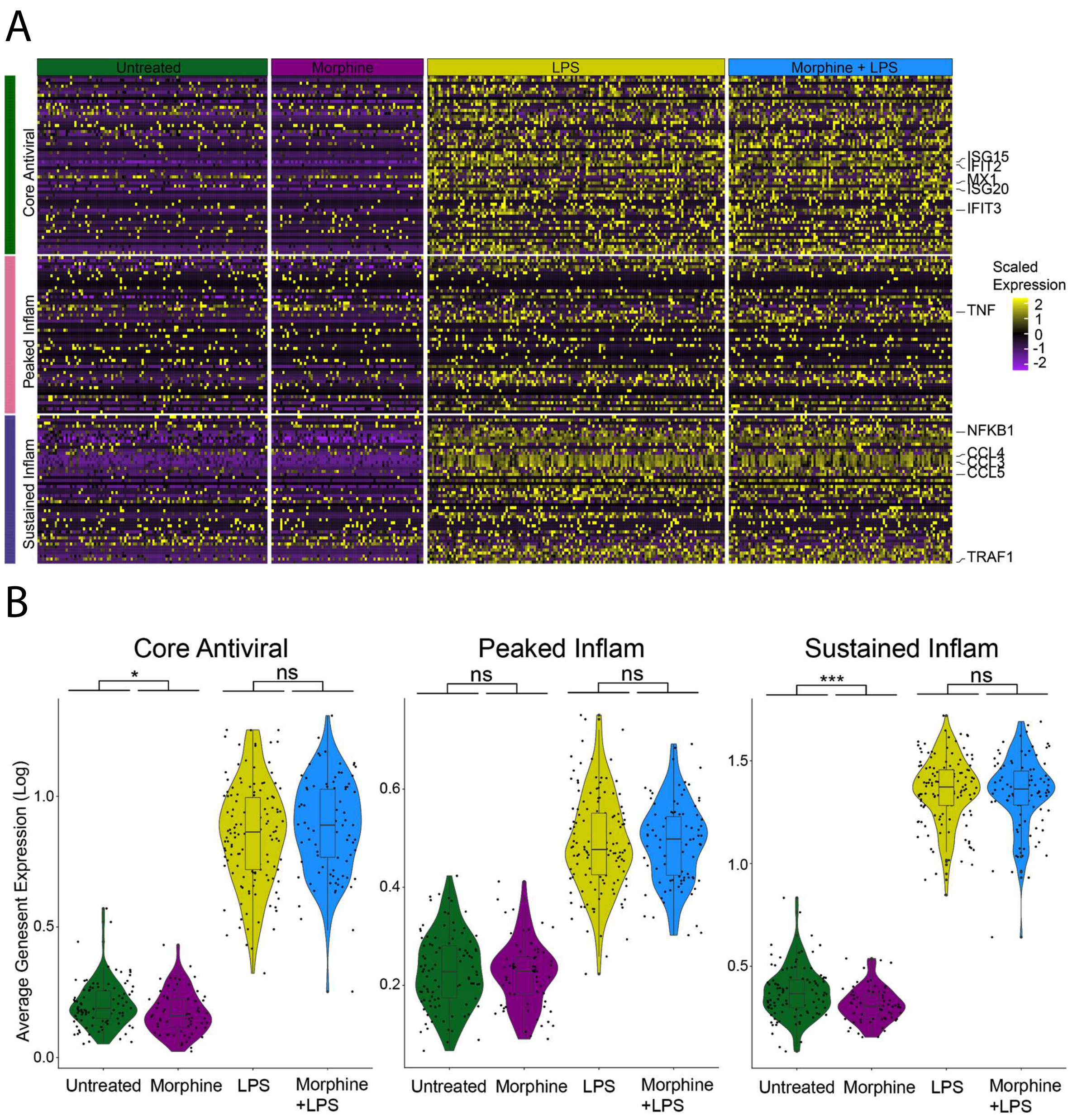

Supplementary Figure 21. Single cell gene expression of antiviral and inflammatory gene modules in in vitro treated Monocytes. a, Heatmap of scaled expression of core antiviral, peaked inflammatory, and sustained inflammatory gene modules observed across all naive state samples and LPS treated samples . **b,** Average expression of all genes in each gene set (log expression) for each cell, grouped naive state samples (Untreated (106 cells), Morphine (70 cells)) and LPS treated samples (LPS (137 cells), Morphine+LPS (103 cells)). Inset box plots show the median, lower and upper hinges that correspond to the first quartile (25th percentile) and third quartile (75th percentile), and the upper and lower whiskers extend from the smallest and largest hinges at most 1.5 times the interquartile range. Two-tailed T-test with comparison tests between control and morphine treated groups for each gene set.: For core antiviral gene set expression: comparison tests between Untreated and Morphine ($p=0.013$), as well as LPS and Morphine+LPS ($p=0.22$). For peaked inflammatory gene set expression: comparison tests between Untreated and Morphine ($p=0.64$), as well as LPS and Morphine+LPS ($p=0.84$). For sustained inflammatory gene set expression: comparison tests between Untreated and Morphine ($p=0.00016$), as well as LPS and Morphine+LPS ($p=0.63$). *$p < 0.05$, **$p < 0.01$, ***$p < 0.001$, ****$p < 0.0001$.

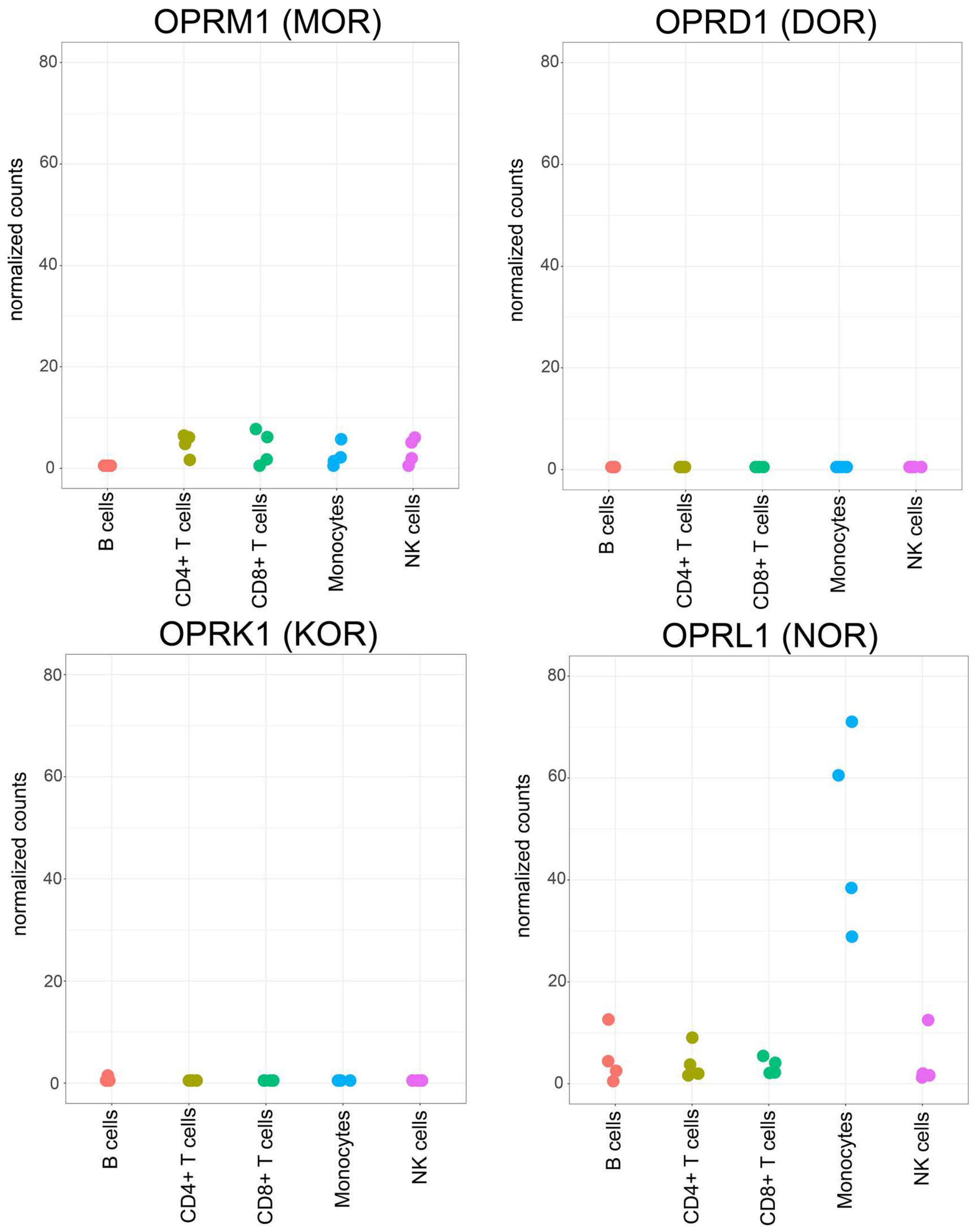

Supplementary Figure 22. Opioid receptor gene expression in peripheral blood immune cell populations from bulk RNA-seq. Bulk RNA-seq data from Corces et al. 2016[28] was reanalyzed. Normalized expression of opioid receptor genes MOR (OPRM1), DOR (OPRD1), KOR (OPRK1), and NOR (OPRL1) in healthy control donors across immune cell types: B cells, CD4+ T cells, CD8+ T cells, Monocytes, NK cells. Source data listing genes and expression values are provided in Source Data file.

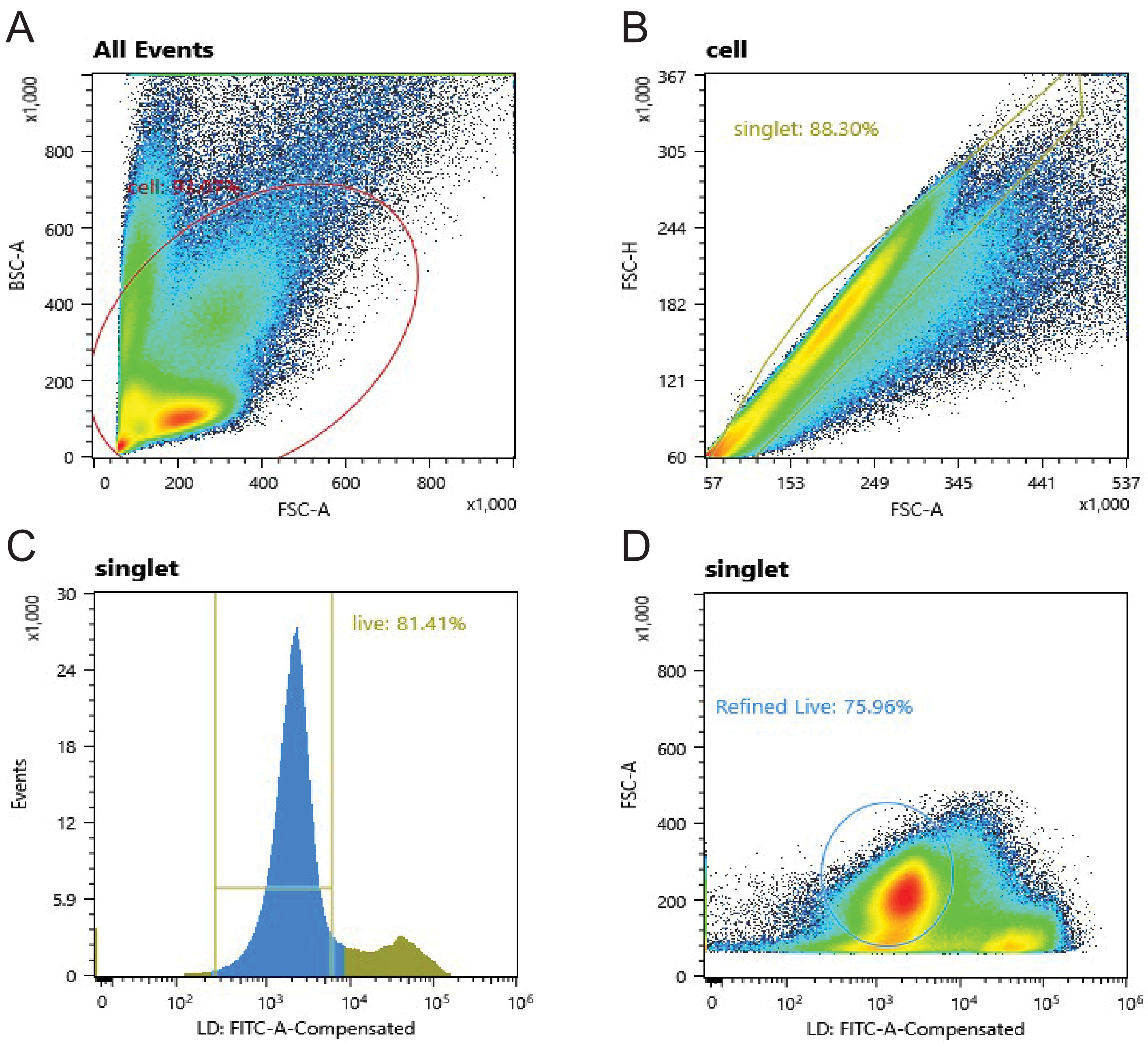

Supplementary Figure 23. FACS gating strategy for isolation of live PBMC. a, The region corresponding with putative cells was selected on the forward-scatter (FSC) vs back-scatter (BSC) plot as the first gate. b, Singlets were then selected along a roughly linear region on a FSC (height) vs FSC (area) plot derived from the “cell” gate. c, A histogram showing number of events by fluorescence intensity for cells within the “singlet” gate. The blue region represents sorted cells. d, Fluorescence intensity was plotted against FSC (area) to select live cells in a more refined way. The “Refined Live” gate was used to sort live cells.

Core Antiviral			Peaked Inflammatory			Sustained Inflammatory		
IL15RA	SLCO3A1	DHX58	ORAI2	PDE4B	PSTPIP2	TLR1	LMO4	MARCKSL1
SAMD9L	OAS2	NLRC5	TNFAIP2	SOCS3	INSIG1	BCL2A1	ACSL1	PIK3R5
PTTG1	STAT2	SP140	MALT1	RALGDS	RASGEF1B	TAGAP	CD200	SQSTM1
ZNFX1	PML	SLFN5	CD44	BCL2L11	DDHD1	MTPN	NAMPT	GADD45B
NT5C3A	ZBP1	IFIT3	LCP2	NFKBIE	ICOSLG	NFKB2	RNF19B	SLC2A6
IRF7	IFIH1	XAF1	RFFL	TSHZ1	ARG2	NFKB1	PTPRJ	IL6
IFITM2	RSAD2	CD69	NFKBID	RASA2	PTPRE	CFLAR	IL1B	PARP14
IFITM1	OAS3	DAXX	CLCN7	MCOLN2	NUP54	C15orf48	SBDS	TXNRD1
IFITM3	ISG15	PARP9	PLK2	SLC25A25	TNFAIP3	SOD2	CXCL16	TRAF1
PARP12	IFIT2	RTP4	LZTFL1	RBM7	PIP5K1A	CLIC4	SLFN12L	
RNASET2	GBP3	DTX3L	LDLR	TRIM13	TNIP1	ST3GAL5	RHBDF2	
TAP1	OAS1	TOR3A	SLC25A37	ARHGEF3	MPP5	TANK	KPNA3	
STAT1	GBP2	DDX58	KLF7	DUSP16		RAB10	GBP5	
EIF2AK2	MNDA	UBA7	NFKBIA	PLAGL2		CCL4	EHD1	
USP18	HERC6	IFI44	SPATA13	PLEKHO2		CCL4L2	ITGA5	
DDX60	MX1	MOV10	CPD	SERTAD2		CCL3	B3GNT2	
MITD1	ETNK1	CXCL10	ZEB2	PLSCR1		CCL3L3	SH3BP5	
USP25	ISG20	OASL	NCK1	SLC16A10		CD40	ELL2	
BST2	TRIM5	ADAR	TNF	RELA		TNFRSF1B	PLEK	
IFI35	CMPK2		TOP1	NFKBIZ		CCL5	MAPKAPK2	

Supplementary Table 1. Gene lists for antiviral inflammatory gene modules in PBMC. List of genes assayed in each module. Core antiviral module = 59 genes. Peaked inflammatory module = 52 genes. Sustained inflammatory module = 49 genes.

Reagent/Material	Vendor	Cat#
LIVE/DEAD Fixable Green Cell Stain Kit, for 488nm Excitation	Thermo Fisher	L34969
RPMI 1640 Medium, No Glutamine	Thermo Fisher	21870076
Fetal Bovine Serum, heat inactivated, qualified, OneShot, USDA Approved	Thermo Fisher	A3840202
L-Glutamine (200mM)	Thermo Fisher	25030149
MEM Non Essential Amino Acids Solution (100X)	Thermo Fisher	11-40-050
HEPES (1M)	Thermo Fisher	15-630-106
Sodium Pyruvate (100mM)	Thermo Fisher	11360070
Gibco 2-Mercaptoethanol	Thermo Fisher	21985023
LPS-EK Ultrapure	Invivogen	tlrl-peklps
Chromium Single Cell 3′ Library and Gel Bead Kit	10X Genomics	PN-120237
Dynabeads MyONE Silane	Thermo Fisher	37002D
SPRIselect	Beckman-Coulter	B23317
Bioanalyzer High-Sensitivity DNA Kit	Agilent	5067-4626
NextSeq Reagent Cartridge	Illumina	
NextSeq Buffer Cartridge	Illumina	
NextSeq HB (?) buffer	Illumina	
NextSeq Flow Cell	Illumina	
FicollPaque PLUS	GE Healthcare	17-1440
Morphine Sulfate	Sigma Aldritch	M8777-25G
ZymoPure Quick RNA Miniprep Kit	Zymo Research	R1055
SuperScript IV First-Strand Synthesis System	Thermo Fisher	18091200
PowerUp SYBR Green Master Mix	Thermo Fisher	A25742
Cell Staining Buffer	BioLegend	420201
Human TruStain FcX (Fc Receptor Blocking Solution)	BioLegend	422301
Flowmi Cell Strainers	Bel-Art	H13680-0040

Supplementary Table 2. Reagents and materials. All reagents and materials used in this study.

Primer Name	Vendor	Sequence
ActB For	IDT	CATGTACGTTGCTATCCAGGC
ActB Rev	IDT	CTCCTTAATGTCACGCACGAT
ISG15 For	IDT	CAGCCATGGGCTGGGAC
ISG15 Rev	IDT	CTTGATCCTGCTCGGATGCT

Supplementary Table 3. RT-qPCR primers. Primers used for RT-qPCR analysis of opioid exposed PBMC.

HTO barcode	Sample
GTCAACTCTTTAGCG	IFNb_C1
GTCAACTCTTTAGCG	IFNb_C2
GTCAACTCTTTAGCG	IFNb_C3
GTCAACTCTTTAGCG	IFNb_O1
GTCAACTCTTTAGCG	IFNb_O2
GTCAACTCTTTAGCG	IFNb_O3

Supplementary Table 4. Ex-vivo PBMC Hashtag Oligonucleotide (HTO) barcode identities. Oligonucleotide barcodes used for the identification of a transcripts sample of origin in multiplexed scRNA-seq experiments using PBMC from opioid dependent individuals and neighborhood controls.

HTO barcode	Sample
GTCAACTCTTTAGCG	Mock
AGTAAGTTCAGCGTA	+Mor
TGTCTTTCCTGCCAG	Mock+LPS
ATTGACCCGCGTTAG	+Mor+LPS

Supplementary Table 5. In-vitro PBMC Hashtag Oligonucleotide (HTO) barcode identities. Oligonucleotide barcodes used for the identification of a transcripts sample of origin in multiplexed scRNA-seq experiments using PBMC treated with morphine in-vitro.

						1:Absent	2:Present						
**Patient**	**ID**	**Stimulation**	**Sex**	**Age**	**HCV status**	**candep_dsm4r**	**stimdep_dsm4r**	**seddep_dsm4r**	**cocdep_dsm4r**	**alcdep_dsm4r**	**nicdep_dsm4r**	**major depressive disorder**	**ptsd**
Control 1	341601_RN	Naïve	m	45	-	2	2	1	1	2	2	2	1
Control 2	383301_RN	Naïve	m	41	-	1	2	1	1	2	1	2	2
Control 3	265501_RN	Naïve	m	26	-	2	2	1	1	2	1	1	1
Control 4	262001_RN	Naïve	m	24	-	2	2	1	1	1	2	2	2
Control 5	259901_RN	Naïve	m	33	-	1	1	1	1	1	1	1	1
Control 6	278201_RN	Naïve	f	41	-	2	1	1	1	2	2	2	1
Control 7	370701_RN	Naïve	f	45	-	1	1	1	1	1	2	1	2
Control 1 LPS	265501_RL	LPS	m	26	-	2	2	1	1	2	1	1	1
Control 2 LPS	262001_RL	LPS	m	24	-	2	2	1	1	1	2	2	2
Control 3 LPS	259901_RL	LPS	m	33	-	1	1	1	1	1	1	1	1
Opioid 1	304901_RN	Naïve	m	45	+	1	2	1	2	1	2	2	1
Opioid 2	333601_RN	Naïve	m	41	+	2	1	1	2	2	2	1	1
Opioid 3	339001_RN	Naïve	m	26	+	2	2	1	2	1	2	2	2
Opioid 4	267001_RN	Naïve	m	24	-	2	1	1	1	1	1	2	2
Opioid 5	287801_RN	Naïve	m	33	-	1	2	1	2	2	2	2	1
Opioid 6	270801_RN	Naïve	f	41	+	2	2	2	1	2	2	2	2
Opioid 7	368401_RN	Naïve	f	45	+	1	2	2	1	2	2	2	2
Opioid 1 LPS	339001_RL	LPS	m	26	+	2	2	1	2	1	2	2	2
Opioid 2 LPS	267001_RL	LPS	m	24	-	2	1	1	1	1	1	2	2
Opioid 3 LPS	287801_RL	LPS	m	33	-	1	2	1	2	2	2	2	1

Supplementary Table 6. Patient sample demographics and comorbidities for Opioid dependency cohort and controls stimulated with LPS. Sample ID's, treatment groups, demographics information and comorbidity status for patients included in the LPS stimulation scRNA-seq experiment. Comorbidity status is given for the following: Hepatitis C infection (HCV status), cannabis dependency (candep_dsm4r), stimulant dependency (stimdep_dsm4r), sedative dependency (seddep_dsm4r), cocaine dependency (cocdep_dsm4r), alcohol dependency (alcdep_dsm4r), nicotine dependency (nicdep_dsm4r), major depressive disorder, and post-traumatic stress disorder (PTSD).

								1:Absent	2:Present						
Patient	ID	RNA_ID	Hashtag_ID	Stimulation	Sex	Age	HCV status	candep_dsm4r	stimdep_dsm4r	seddep_dsm4r	cocdep_dsm4r	alcdep_dsm4r	nicdep_dsm4r	major depressive disorder	ptsd
Control 1	289101	RNA_RI	Hashtag_RI	IFNb	m	44	-	1	1	1	1	1	1	1	2
Control 2	264401	RNA_RI	Hashtag_RI	IFNb	m	46	-	1	1	1	1	1	1	2	1
Control 3	354701	RNA_RI	Hashtag_RI	IFNb	f	47	-	1	1	1	1	1	1	1	2
Opioid 1	353601	RNA_RI	Hashtag_RI	IFNb	m	44	-	2	2	2	2	2	1	2	1
Opioid 2	296001	RNA_RI	Hashtag_RI	IFNb	m	46	-	2	2	2	2	1	2	2	2
Opioid 3	312501	RNA_RI	Hashtag_RI	IFNb	f	47	-	2	1	1	1	1	1	2	2

Supplementary Table 7. Patient sample demographics and comorbidities for Opioid dependency cohort and controls stimulated with IFNβ. Sample ID’s, treatment groups, demographics information and comorbidity status for patients included in the IFNβ stimulation scRNA-seq experiment. Comorbidity status is given for the following: Hepatitis C infection (HCV status), cannabis dependency (candep_dsm4r), stimulant dependency (stimdep_dsm4r), sedative dependency (seddep_dsm4r), cocaine dependency (cocdep_dsm4r), alcohol dependency (alcdep_dsm4r), nicotine dependency (nicdep_dsm4r), major depressive disorder, and post-traumatic stress disorder (PTSD).
