# Supplementary material for: Single cell transcriptomics reveals opioid usage evokes widespread suppression of antiviral gene program
Source: Nat Commun. 2020 May 26;11:2611. doi: 10.1038/s41467-020-16159-y (PMC7250875; doi:10.1038/s41467-020-16159-y)
Supplement: Supplementary file 3 — Reporting Summary [file 41467_2020_16159_MOESM3_ESM.pdf]

nature research

Corresponding author(s): Christine S. Cheng

Last updated by author(s): Nov 15, 2019

Reporting Summary

Nature Research wishes to improve the reproducibility of the work that we publish. This form provides structure for consistency and transparency in reporting. For further information on Nature Research policies, see Authors & Referees and the Editorial Policy Checklist.

Statistics

For all statistical analyses, confirm that the following items are present in the figure legend, table legend, main text, or Methods section.

n/a	Confirmed	
☐	☒	The exact sample size (*n*) for each experimental group/condition, given as a discrete number and unit of measurement
☐	☒	A statement on whether measurements were taken from distinct samples or whether the same sample was measured repeatedly
☐	☒	The statistical test(s) used AND whether they are one- or two-sided<br>*Only common tests should be described solely by name; describe more complex techniques in the Methods section.*
☐	☒	A description of all covariates tested
☐	☒	A description of any assumptions or corrections, such as tests of normality and adjustment for multiple comparisons
☐	☒	A full description of the statistical parameters including central tendency (e.g. means) or other basic estimates (e.g. regression coefficient) AND variation (e.g. standard deviation) or associated estimates of uncertainty (e.g. confidence intervals)
☐	☒	For null hypothesis testing, the test statistic (e.g. *F*, *t*, *r*) with confidence intervals, effect sizes, degrees of freedom and *P* value noted<br>*Give P values as exact values whenever suitable.*
☒	☐	For Bayesian analysis, information on the choice of priors and Markov chain Monte Carlo settings
☒	☐	For hierarchical and complex designs, identification of the appropriate level for tests and full reporting of outcomes
☒	☐	Estimates of effect sizes (e.g. Cohen's *d*, Pearson's *r*), indicating how they were calculated

Our web collection on statistics for biologists contains articles on many of the points above.

Software and code

Policy information about availability of computer code

Data collection	10X Chromium V2
Data analysis	R v3; cellRanger v2.1.0 and v.3.0.2; bcl2fastq v2.18; STAR v.2.5.1a; Metascape (https://metascape.org/gp/index.html#/main/step1); R packages(Seurat v2.3.4; ComplexHeatmap v1.20.0; circlize v.0.4.6; MAST v1.10.0; ggplot2 v3.1.1)

For manuscripts utilizing custom algorithms or software that are central to the research but not yet described in published literature, software must be made available to editors/reviewers. We strongly encourage code deposition in a community repository (e.g. GitHub). See the Nature Research guidelines for submitting code & software for further information.

Data

Policy information about availability of data

All manuscripts must include a data availability statement. This statement should provide the following information, where applicable:

- Accession codes, unique identifiers, or web links for publicly available datasets
- A list of figures that have associated raw data
- A description of any restrictions on data availability

Processed cell hashing scRNA-seq data of in vitro morphine treated PBMCs is available from GEO under accession GSE128879. Raw and processed scRNA-seq data of opioid-dependent individuals and neighborhood controls is available from dbGaP under accession phs000277.v2.p1 at https://www.ncbi.nlm.nih.gov/projects/gap/cgi-bin/study.cgi?study_id=phs000277.v2.p1. The processed scRNA-seq data of LPS-treated PBMCs of opioid-dependent individuals and controls is available from single cell portal to view and download at https://singlecell.broadinstitute.org/single_cell/study/SCP587/. The processed scRNA-seq data of IFNb-treated PBMCs of opioid-dependent individuals and controls is available from single cell portal to view and download at https://singlecell.broadinstitute.org/single_cell/study/SCP589/. The processed scRNA-seq data of in vitro morphine treated PBMCs is available from single cell portal to view and download at https://singlecell.broadinstitute.org/single_cell/study/SCP591/. All the other data supporting the findings of this study are available within the article and its supplementary information files and from the corresponding authors upon reasonable request

Field-specific reporting

Please select the one below that is the best fit for your research. If you are not sure, read the appropriate sections before making your selection.

☒ Life sciences ☐ Behavioural & social sciences ☐ Ecological, evolutionary & environmental sciences

For a reference copy of the document with all sections, see nature.com/documents/nr-reporting-summary-flat.pdf

Life sciences study design

All studies must disclose on these points even when the disclosure is negative.

Sample size	To estimate the number of necessary individuals to have enough statistical power for scRNAseq, we performed a simulation based statistical power analysis using powersimR (Vieth et al., 2017). With a false discovery rate (FDR) of 0.05 and true positive rate (TPR) of 0.8, the projected sample size needed with this effect size is approximately between 5 to 6 individuals. Sample size for scRNA-seq experiments were chosen based on the power analysis (7 opioid dependent individuals vs 7 controls).
Data exclusions	No data excluded.
Replication	The first level of replication we used is the biological replicates of multiple patients per condition. Also, for this type of study (scRNA-seq) each single cell transcriptome can be considered a sequencing result. Therefore the individual cells for each cell type can be considered a secondary biological replicate. Due to sample scarcity we were not able to perform technical replicates using these specific samples, however our results were consistent over multiple experiments using different patient samples.
Randomization	Allocation was not random and was chosen specifically based on whether patient samples came from opioid-exposed or non-opioid exposed individuals. We controlled for age, sex, and ethnicity by matching samples according to these variables.
Blinding	Given that the "treatment" group for this study (opioid users) was self selected, and the fact that our bioinformatic analysis strategy was inherently unbiased, blinding was not necessary/possible for this study.

Reporting for specific materials, systems and methods

We require information from authors about some types of materials, experimental systems and methods used in many studies. Here, indicate whether each material, system or method listed is relevant to your study. If you are not sure if a list item applies to your research, read the appropriate section before selecting a response.

Materials & experimental systems

n/a	Involved in the study	
☐	☒	Antibodies
☒	☐	Eukaryotic cell lines
☒	☐	Palaeontology
☒	☐	Animals and other organisms
☒	☐	Human research participants
☒	☐	Clinical data

Methods

n/a	Involved in the study	
☒	☐	ChIP-seq
☒	☐	Flow cytometry
☒	☐	MRI-based neuroimaging

Antibodies

Antibodies used	TotalSeq™-A0251 anti-human Hashtag 1 Antibody, Cat#394601, Lot#B260326, Clone LNH-94 2M2, BioLegend<br>TotalSeq™-A0252 anti-human Hashtag 2 Antibody, Cat#394603, Lot#B260322, Clone LNH-94 2M2, BioLegend<br>TotalSeq™-A0253 anti-human Hashtag 3 Antibody, Cat#394605, Lot#B260318, Clone LNH-94 2M2, BioLegend<br>TotalSeq™-A0254 anti-human Hashtag 4 Antibody, Cat#394607, Lot#B260315, Clone LNH-94 2M2, BioLegend<br>TotalSeq™-A0255 anti-human Hashtag 5 Antibody, Cat#394609, Lot#B260314, Clone LNH-94 2M2, BioLegend<br>TotalSeq™-A0256 anti-human Hashtag 6 Antibody, Cat#394611, Lot#B260341, Clone LNH-94 2M2, BioLegend<br>TotalSeq™-A0257 anti-human Hashtag 7 Antibody, Cat#394613, Lot#B260339, Clone LNH-94 2M2, BioLegend<br>TotalSeq™-A0258 anti-human Hashtag 8 Antibody, Cat#394615, Lot#B260336, Clone LNH-94 2M2, BioLegend<br>TotalSeq™-A0259 anti-human Hashtag 9 Antibody, Cat#394617, Lot#B260333, Clone LNH-94 2M2, BioLegend<br>TotalSeq™-A0260 anti-human Hashtag 10 Antibody, Cat#394619, Lot#B260330, Clone LNH-94 2M2, BioLegend<br>TotalSeq™-A0262 anti-human Hashtag 12 Antibody, Cat#394623, Lot#B264718, Clone LNH-94 2M2, BioLegend<br>TotalSeq™-A0263 anti-human Hashtag 13 Antibody, Cat#394625, Lot#B264717, Clone LNH-94 2M2, BioLegend
Validation	As delineated in the technical data sheets for these antibodies, BioLegend validates all antibodies using immunofluorescent staining and Flow Cytometry, while the conjugated oligo identities were confirmed by sequencing. In the hands of the researchers, antibody efficacy in cell hashing was determined using the BioLegend cell hashing protocol with healthy, non-opioid-exposed, bulk PBMC. Quality/efficacy of cell hashing was evaluated by comparing the expected numbers of cells targeted per antibody hash to the number of cells detected following 10X scRNA-seq.
